# Exploring metabolic adaptation of *Streptococcus pneumoniae* to antibiotics

**DOI:** 10.1038/s41429-020-0296-3

**Published:** 2020-03-24

**Authors:** Anne Leonard, Kevin Möhlis, Rabea Schlüter, Edward Taylor, Michael Lalk, Karen Methling

**Affiliations:** 1 Institute for Biochemistry, Metabolomics, University of Greifswald, Felix-Hausdorff-Str. 4, 17489 Greifswald, Germany; 2 Imaging Center of the Department of Biology, University of Greifswald, F.-L-Jahn-Str. 15, 17489 Greifswald, Germany; 3 University of Lincoln, School of Life Sciences, Green Lane, LN67DL Lincoln, England United Kingdom

**Keywords:** Metabolomics, Bacterial infection

## Abstract

The Gram-positive bacterium *Streptococcus pneumoniae* is one of the common causes of community acquired pneumonia, meningitis, and otitis media. Analyzing the metabolic adaptation toward environmental stress conditions improves our understanding of its pathophysiology and its dependency on host-derived nutrients. In this study, extra- and intracellular metabolic profiles were evaluated to investigate the impact of antimicrobial compounds targeting different pathways of the metabolome of *S. pneumoniae* TIGR4*Δcps*. For the metabolomics approach, we analyzed the complex variety of metabolites by using ^1^H NMR, HPLC-MS, and GC–MS as different analytical techniques. Through this combination, we detected nearly 120 metabolites. For each antimicrobial compound, individual metabolic effects were detected that often comprised global biosynthetic pathways. Cefotaxime altered amino acids metabolism and carbon metabolism. The purine and pyrimidine metabolic pathways were mostly affected by moxifloxacin treatment. The combination of cefotaxime and azithromycin intensified the stress response compared with the use of the single antibiotic. However, we observed that three cell wall metabolites were altered only by treatment with the combination of the two antibiotics. Only moxifloxacin stress-induced alternation in CDP-ribitol concentration. Teixobactin-Arg10 resulted in global changes of pneumococcal metabolism. To meet the growing requirements for new antibiotics, our metabolomics approach has shown to be a promising complement to other OMICs investigations allowing insights into the mode of action of novel antimicrobial compounds.

## Introduction

In community-acquired pneumonia (CAP), a serious illness associated with morbidity and mortality [1–4], the most frequent pathogen isolated in adults is *Streptococcus pneumoniae* (the pneumococcus) [5, 6]. *S. pneumoniae*, a Gram-positive facultative pathobiont, commonly inhabits the upper respiratory tract of humans and resulting in at least 1–2 million infant deaths every year worldwide [4, 7, 8]. The pneumococcus is a fermentative bacterium with a high rate of glycolysis [9] and lacks of the Entner-Doudoroff pathway, aerobic tricarboxylic acid cycle, and the electron transport chain of aerobe and anaerobe respiration [9–11]. A challenge in treating pneumococcal infections is the increasing drug resistance over recent decades. Currently, 15–30% of the pneumococcal strains are classified as multi drug resistant [12]. Due to the side effects of fluoroquinolones the European Medicines Agency for Europe has emulated the U.S. Food and Drug Administration in introducing restrictions on their use. Typical antibiotic therapies of pneumococcus include *β*-lactams, macrolides, or fluoroquinolones alone or in combination. The use of combination therapy of two antibiotics has been shown to achieve superior outcome compared with monotherapy [13–16].

Moxifloxacin is a recently-developed fluoroquinolone that acts by binding to the topoisomerase enzymes II (DNA gyrase) thus preventing replication, transcription, and repair of bacterial DNA [17]. *S. pneumoniae* appears to be less resistant to 8-methoxy quinolone moxifloxacin compared with earlier fluoroquinolones [18].

The cephalosporins are a group of *β*-lactam antibiotics. With a mode of action comparable to penicillin, inhibiting cell wall synthesis and modification by binding penicillin-binding proteins (PBPs), this results in arrest of cell growth and eventual cell lysis of bacteria. PBPs are membrane-associated enzymes involved in the final step of peptidoglycan assembly and turnover [19]. Penicillin allergy is the most common drug allergy concerning up to 15% of hospitalized patients, which can present a barrier to treatment of *S. pneumoniae* infections [20]. Cephalosporines like cefotaxime were developed to face bacteria resistant to penicillin or treat infections in humans allergic to penicillin [21]. Cefotaxime is rapidly deacetylated in the body to desacetyl cefotaxime, which has a similar antimicrobial spectrum to cefotaxime [21].

Azithromycin is a macrolide derivative of erythromycin. The azolide antimicrobial agent is active against pathogens responsible for infections of the respiratory tract, skin, and soft tissues in human [22]. Azithromycin inhibits bacterial growth and replication by interrupting protein biosynthesis [23].

*S. pneumoniae* has repeatedly been shown to be capable of rapidly developing or acquiring resistance to the commonly used agents of treating pneumonia. The use of antibiotic combinations increases the spectrum of targeted bacterial species in addition to increased efficacy, limiting the occurrence and spread of resistant bacterial populations. Many studies focused on the combination of a cephalosporin and a macrolide [6, 15, 16, 24, 25].

The rapid development of bacterial resistance to antibiotics is one of the most recent threats to human health [26]. In the last 40 years only two new classes of antibiotics have been discovered [27]. In 2015, the organism, *Eleftheria terrae*, was found to produce a novel depsipeptide antibiotic, called teixobactin [28]. The antibiotic kills a broad range of Gram-positive bacteria including multi drug resistance strains [28]. There is currently no evidence of acquired resistance. Teixobactin binds to the pyrophosphate motifs of multiple bacterial cell-wall substrates such as lipid II (precursor of peptidoglycan) and lipid III (precursor of cell-wall teichoic acid) [28]. Teixobactin is a naturally occurring molecule and has a number of issues preventing direct introduction to the clinic [28, 29]. Parmar et al. designed a synthesis of teixobactin analogs that sacrifices antibiotic activity for ease of synthesis through the replacement of the difficulty to synthesize enduracididine with arginine [29].

Understanding the metabolism of pneumococci is essential to gain insights into the adaptation strategies that are required to deal with the host environment during infection and to identify new drug targets [30]. We analyzed the metabolic response of *S. pneumoniae* to limited growth conditions as well as during treatment with antimicrobial compounds to identify metabolic adaptation processes. This study will contribute to a better understanding of pneumococcal physiology. We have used our recently established workflow [9] to analyze the alterations of extra- and intracellular metabolites of *S. pneumoniae* after treatment with different antimicrobial compounds. To cover a broad range of metabolic adaptations, pneumococci were exposed to three commonly used antibiotics with different targets of action (cefotaxime, azithromycin, and moxifloxacin), a combination of two antibiotics (cefotaxime and azithromycin), and the new antimicrobial compound (teixobactin-Arg 10 [29]).

## Material and methods

### Bacterial strain and growth conditions

The non-encapsulated *S. pneumoniae* TIGR4*Δcps* used as model organism was cultivated in the chemically-defined medium RPMI_modi_ 1640 (HyClone) [9] and grown on Columbia blood agar plates (Oxoid) in the presence of the appropriate antibiotic (150 µg ml^−1^ kanamycin). The cultivation was performed as described previously [9]. At a mid-exponential phase OD_600_ of 0.5 the bacterial cells were treated with either 0.005 µg ml^−1^ (0.5 × minimal inhibitor concentration (MIC)) cefotaxime (Sigma-Aldrich), 0.064 µg ml^−1^ (2 × MIC) azithromycin (Sigma-Aldrich), 0.8 µg ml^−1^ (2 × MIC) moxifloxacin (Sigma-Aldrich), 2 µg ml^−1^ teixobactin-Arg10 (cooperation with University of Lincoln, School of Life Sciences [31, 32]), and combination of 0.5 × MIC cefotaxime and 2 × MIC azithromycin for 90 min. For the control, bacterial cells were cultivated without antibiotic. We obtained five independent biological replicates for the metabolome analysis with exception of teixobactin analog (four independent biological replicates). Extra- and intracellular metabolome samples were taken at 15, 30, 60, and 90 min (t_15_, t_30_, t_60_, and t_90_) after the addition of each antimicrobial compound.

### Minimal inhibitory concentration (MIC)

MIC is defined as lowest concentration of a compound/drug/antibiotic preventing visible growth of a microorganism [33]. The MIC for each antibiotic compounds was determined for *S. pneumoniae* TIGR4*Δcps* cultivated in modified RPMI medium (*n* **=** 3). The MIC determination for teixobactin analog was tested only once due to available restricted amount of compound. At an OD_600_ of 0.1 each antibiotic was added to the bacterial main culture in dilution series. At every hour, the OD_600_ was measured for 6 h.

### Preparation and ^1^H NMR spectroscopic analysis of extracellular metabolites

For the analysis of extracellular metabolites, 2 ml bacterial cell suspension was filtered and analyzed by using ^1^H Nuclear Magnetic Resonance (^1^H NMR) spectroscopy as described previously [9].

### Preparation of intracellular metabolite extracts

The sampling of intracellular metabolites was described earlier [9]. In brief, 15 OD units (1 OD units equates 1 ml at OD_600nm_ of 1) were sampled by using the vacuum-dependent fast-filtration approach. The filtered cells were washed twice with 5 ml cold isotonic sodium chloride solution (130 mM). After the transfer of the filter into extraction solution (60% ethanol [w/v]), the samples were shock frozen (liquid N_2_) and stored at −80 °C prior extraction. To obtain the intracellular metabolite extracts, the bacterial cells were washed off the filter by shaking and vortexing. Resuspended bacteria were transferred into a 50 ml tube containing glass beads with 0.1 mm diameter (Sartorius AG). The internal standards (see Supplementary Table S1) for LC-MS and GC-MS analyses were added. Two cell disruption cycles (2 × 40 s, 6.0 m/s) were performed by using the FastPrep-24 instrument (MP Biomedicals). The transfer, washing, and centrifugation of the bacterial cell extract were described earlier [9]. The supernatant was diluted with water and stored at −80 °C for lyophilization. The dried samples were resuspended in 1.5 ml cold water and divided into two equal parts. One part of each sample was directly frozen and lyophilized for further GC-MS analysis. 150 µl ice-cold trichloromethane was added to the other part of the samples. After shaking and vortexing for ten times the samples were stored at −20 °C for 5 min and centrifuged at 4 °C and 13,000 rpm for 5 min. The upper layer of each sample was collected, frozen, and lyophilized for LC-MS analysis.

### HPLC-MS and GC-MS analyses

The measurements of the lyophilized extracts with LC-MS and GC-MS according to the protocol are described elsewhere [9].

The qualitative and quantitative analyses of LC-MS data were carried out by using DataAnalysis v4.0 and QuantAnalysis v2.0 software (Bruker Daltonik GmbH). Absolute concentrations of metabolites were determined using calibration curves for each metabolite (see Supplementary Table S2). Signals detected in blank samples were excluded from the data analysis of biological samples.

For the GC-MS analysis, the identification and quantification of intracellular metabolites were performed using MassHunter (Agilent). Identification of peaks was carried out by comparison of retention time and mass spectra with those of standard compounds in a database with a similarity of 75% or higher. For quantification, the areas of the identified peaks were normalized to the area of peaks of internal standard compounds (see Supplementary Table S2). This ratio represented the relative amount of each metabolite. Absolute concentrations of metabolites were determined using calibration curves for each metabolite. Signals detected in blank samples were excluded from the data analysis of biological samples.

### Determination of the adenylate energy charge

Adenylate energy charge (AEC) was calculated for each sample using the absolute concentrations of AMP, ADP, and ATP [34].

### Colony forming units (CFU)

Pneumococci were cultivated as described in the cultivation section above. At t_30_ and t_90_ after adding antibiotics, 50 μl of each culture was transferred into a 1.5 ml micro-reaction tube that contained 950 μl modified RPMI medium. The tube was gently swung to ensure an equal dilution. The dilution step was repeated four times until a dilution factor of 3.2 × 10^6^ was reached. 50 μl of the final dilution was spread equally with an inoculation spreader on a blood agar plate and incubated for 20 h (37 °C/5% CO_2_). After 20 h, the colonies were counted.

### Transmission electron microscopy

For the transmission electron microscopy (TEM), 15 OD units were transferred into a 50 ml tube at t_90_ and centrifugated carefully (3000 rpm, 20 °C, 3 min). The pellet was washed with 10 ml isotonic NaCl solution and centrifuged again. The cells were fixed with a solution containing 2.5% glutaraldehyde and 2% paraformaldehyde in buffer (100 mM cacodylate buffer, 10 mM CaCl_2_, 10 mM MgCl_2_, 0.09 M sucrose; pH 7) for 20 min on ice and then stored at 4 °C until further processing using two different methods. For method A, cells were embedded in low gelling agarose, post fixed in 1% osmium tetroxide in buffer for 1 h, and then stained en bloc with 2% uranyl acetate in 0.9% sodium chloride for 30 min at room temperature. For method B, cells were treated with 0.5% glutaraldehyde and 1% osmium tetroxide in buffer for 1 h at 4 °C. Subsequent to embedding in low gelling agarose, cells were fixed with 1% osmium tetroxide in buffer for 1 h at room temperature, and then stained en bloc with 0.5% uranyl acetate in 0.9% sodium chloride at 4 °C overnight.

For both methods, specimens were then dehydrated in graded series of ethanol (30–100%) on ice for 30 min each step, and finally the material was stepwise infiltrated with the acrylic resin LR White according to Hammerschmidt et al. [35]. Sections were cut on an ultramicrotome (Reichert Ultracut, Leica UK Ltd, Milton Keynes, UK), stained with 4% aqueous uranyl acetate for 5 min and analyzed with a transmission electron microscope LEO 906 (Carl Zeiss Microscopy GmbH, Oberkochen, Germany). The micrographs were edited by using Adobe Photoshop CS6.

### Statistics and visualization

Statistical analysis and visualization of data were carried out by using the Prism v7 software (GraphPad). The *p* values were calculated based on two-way ANOVA and significance level of 0.05 was corrected for multiple testing by Šidák. Color-coded heat maps were created with MeV (v4.9). The changes in the extracellular metabolome were analyzed by fold change (FC) calculation of concentrations of each metabolite for each time point relative to the control at the corresponding time point and normalization to the optical density measured at the respective sampling time (see Table S3 in Supplementary material). For the intracellular metabolome there was a normalization step in the sampling protocol. Always 15 OD units of bacteria were sampled and divided into equal parts after extraction for analysis by HPLC-MS and GC-MS.

## Results

### Inventory of pneumococcal metabolic profile

**Fig. 1:**
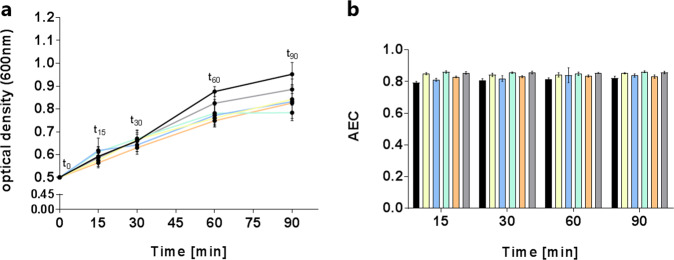
Growth curves (**a**) of *S. pneumoniae* TIGR4*Δcps* in RPMI_modi_ and adenylate energy charges (**b**). Curves and columns are colored according to the antibiotic stresses: control (black), cefotaxime (yellow), azithromycin (blue), combination of cefotaxime and azithromycin (green), moxifloxacin (orange), and teixobactin-Arg10 (gray). Data are shown as mean values ± standard derivation (*n* = 4–5)

To analyze the metabolic adaptation of *S. pneumoniae* TIGR4Δ*cps* to different antimicrobial compounds, bacterial cells were exposed to antibiotics during exponential growth phase. The fast doubling time of the bacteria during this phase ensures a fast turnover of metabolites in the bacteria and thus excellent conditions to study the influence of certain antibiotics on the pneumococcal metabolome. Extra- and intracellular metabolome samples were taken in a time span of 2 h (starting point OD of 0.5) to observe fast and time-resolved alterations. The largest growth difference between control and drug treatment was observed 90 min after adding the antimicrobial compounds (Fig. 1a). In addition to MIC determination (see Fig. S1 in Supplementary material), the CFU were counted (see Fig. S2 in Supplementary material). Furthermore, stressed and control bacteria were examined by transmission electron microscopy at t_90_ after antimicrobial stress, to highlight which morphological variations may be linked to alterations within the metabolome (Fig. S3). The cell wall was only altered in cells stressed by moxifloxacin and teixobactin-Arg10 (Fig. S3). Bacterial cells treated with teixobactin-Arg10 presumably showed a defective cell wall and thus cell components escaped from the cells. In addition, all stressed cells with exception of cells treated with azithromycin showed more and longer white areas inside compared with control. In the control cells and cells stressed with azithromycin the areas were very weak.

The AEC was determined for each sample. In this study, the AEC was 0.84 ± 0.02 for pneumococcal cells under control and stress conditions (Fig. 1b).

Using ^1^H NMR spectroscopy, HPLC-MS and GC-MS, 124 compounds were identified and analyzed (see Supplementary material Tables S3–S6) to cover a broad range of the chemically diverse metabolites. By using ^1^H NMR, 37 metabolites were identified and quantified by using a library of standard spectra. Quantification of 90 metabolites by GC-MS and HPLC-MS was verified by using labeled analytical standard compounds (see Supplementary Table S2). In summary, different metabolic pathways such as glycolysis, the pentose phosphate pathway, peptidoglycan biosynthesis, purine and pyrimidine nucleotide metabolism, cofactor and amino acid metabolism were affected (Table 1). Two kinds of metabolic changes were observable: on the one hand time-dependent metabolic alterations and on the other hand antibiotic stress-dependent changes were visible.

**Table.1:** Table showing numbers of intracellular metabolites significantly changed in amounts (*p* ≤ 0.05)

	Cefotaxime		Azithromycin		Cefotaxime + azithromycin		Moxifloxacin		Teixobactin-Arg10	
	Increased	Decreased	Increased	Decreased	Increased	Decreased	Increased	Decreased	Increased	Decreased
Carbon metabolism	9	5	2	3	13	4	6	4	8	4
Amino acid metabolism	17	2	1	3	18	2	4	2	11	5
Nucleotide metabolism	2	18	0	15	3	18	11	10	2	20
Cell wall precursors	2	4	1	5	4	3	4	2	5	3
Other intermediates	2	2	0	2	3	5	4	1	3	4
Total	63		32		73		48		65	

### Cefotaxime treatment dependent metabolic alterations

**Fig. 2:**
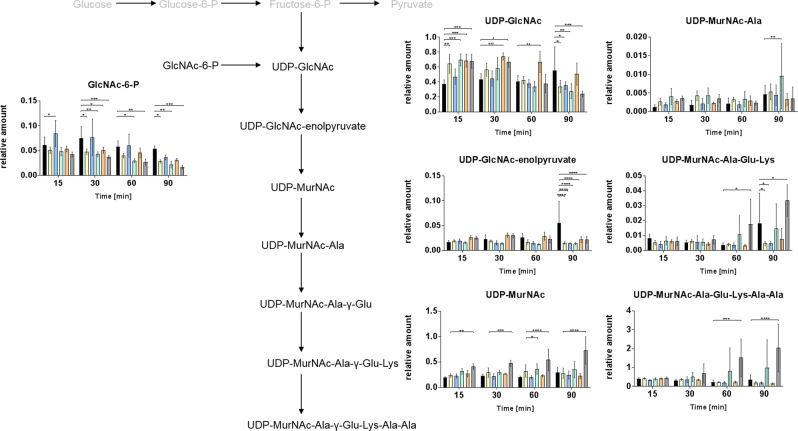
Intermediates of peptidoglycan biosynthesis of *S. pneumoniae* TIGR4*Δcps*. Relative amounts of intracellular metabolites are presented as bar charts. Columns (*n* = 4–5) are colored according to the antibiotic stresses: control (black), cefotaxime (yellow), azithromycin (blue), combination of cefotaxime and azithromycin (green), moxifloxacin (orange), and teixobactin-Arg10 (gray). Asterisks indicate significant differences (*α* = 0.05 after Sidak correction) between the treated cells and the control. *p ≤ 0.05; **p ≤ 0.01; ***p ≤ 0.001; ****p ≤ 0.0001

**Fig. 3:**
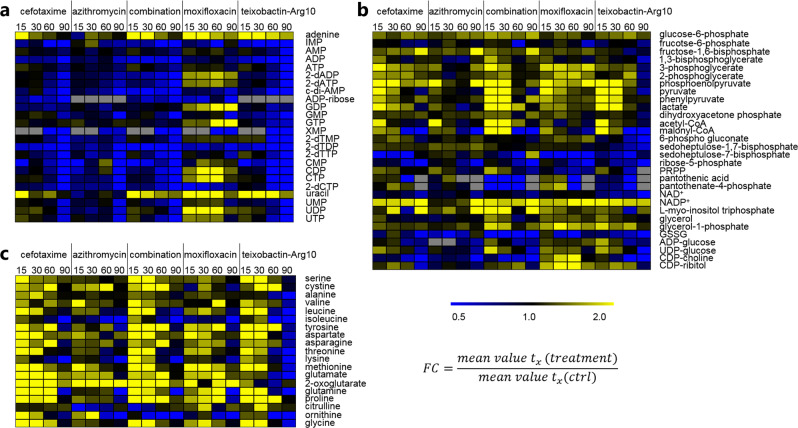
Intracellular metabolites of (**a**) purine and pyrimidine metabolism, (**b**) carbon metabolism, and (**c**) amino acid metabolism of *S. pneumoniae* TIGR4*Δcps*. The illustrated heat maps with fold changes (FC) of metabolite concentrations under conditions of antibiotic treatment referred to control shows metabolites with increased concentrations compared with unstressed bacteria in yellow and with decreased concentrations in blue at the different time points (t_15_–t_90_). Gray fields: metabolites were not detected under stress and/or control conditions

The treatment of pneumococci with cefotaxime changed significantly the levels of 63 intracellular metabolites compared with control conditions. Thereof, 32 metabolites were increased in amount (Table 1). Cefotaxime as inhibitor of cell wall synthesis caused significant decrease in amounts of cell wall precursors UDP-GlcNAc, GlcNAc-6-P, and UDP-MurNAc-Ala-Glu-Lys at t_90_ (Fig. 2). Surprisingly, strong effects of cefotaxime stress were found for glycolysis, amino acid, and nucleotide metabolism. The levels of all glycolytic metabolites following 1,3-bisphosphoglycerate (1,3-bP-glycerate) were enhanced compared with control at t_15_. Increase in amount of pyruvate was also more than twofold, but not significant (*p* > 0.05) (Fig. 3). The enhanced intracellular pyruvate concentration may cause the observed changes in the amount of associated metabolites as observed for alanine as well as acetyl-CoA and lactate (both *p* > 0.05). The intracellular concentration of alanine was increased at t_15_ and t_30_ under stress conditions. Phenylpyruvate and the related metabolite tyrosine showed the same effects intracellularly. Amount of tyrosine increased threefold at t_15_. Also, the amounts of aspartate, asparagine, and threonine were enhanced at t_15_ intracellularly. Moreover, glutamine and related metabolites like glutamate, 2-oxoglutarate and proline, possessed the same intracellular metabolome adaptations with FC ≥ 3 for all metabolites (Fig. 3).

The majority of analyzed nucleotides were significantly decreased in amount intracellularly at t_90_ (Fig. 3), which appears to be a general effect of all stress conditions with exception of DNA gyrase inhibitor moxifloxacin. Only the amount of GTP (*p* > 0.05) showed no changes compared with control conditions. The amounts of GDP and IMP (both *p* > 0.05) decreased compared with control. Twofold higher concentrations were found for the nucleobases adenine and uracil at t_15_ compared with control conditions. In conclusion, intracellular amounts of glycolysis metabolites and amino acids were changed at t_15_ and nucleotides were altered mostly at t_90_ after stress induction.

### Macrolide azithromycin

In general, the treatment with azithromycin caused least metabolic alterations compared with the other tested antibiotics. In total, abundances of 32 metabolites were influenced significantly compared with untreated pneumococci. Although, four intracellular metabolites showed a significant increase and 28 metabolites a decrease in amount (Table 1). Pneumococci stressed with azithromycin showed no significant changes in amino acid metabolome with exception of altered levels of methionine, lysine, and isoleucine, which were influenced the same way by all other antibiotic treatments in this study. 2-Oxoglutarate as intermediate of glutamine/glutamate metabolism was increased two- to five-fold in amount intracellularly at all time points (Fig. 3). Also, glycolysis was less affected compared with other stress conditions. Glycolytic metabolites like fructose-1,6-bisphosphate (frc-1,6-bP) and phosphoenolpyruvate (PEP) showed enhanced concentrations 90 min after adding azithromycin (t_90_) (Fig. 3). Both, pyruvate as end product of glycolysis and the fermentation product lactate were found in similar amounts as in control samples.

The inhibitor of protein synthesis azithromycin decreased significantly the amounts of all detected intracellular purine and pyrimidine metabolites at t_90_ (XMP (*p* > 0.05)) (Fig. 3). Only the purine nucleotides GDP, GTP, and IMP seems to be not affected after azithromycin stress.

### Treatment with the combination of antibiotics—cefotaxime and azithromycin

After pneumococcal exposure to cefotaxime and azithromycin, the intracellular abundances of 73 metabolites were significantly changed compared with control cells. These included 41 increased and 32 decreased metabolites. Bacterial treatment with the combination of the antibiotics induced the largest changes in the metabolome compared with other antimicrobial stress conditions. Surprisingly, seven metabolites showed significant changes only by treatment with combination of the two antibiotics azithromycin and cefotaxime and not by single usage: threonine (t_90_), 4-P-pantothenate (t_15_ and t_30_), acetyl-CoA (t_15_–t_60_), malonyl-CoA (t_15_), glutathione disulfide (GSSG) (t_15_), UDP-MurNAc (t_60_), and UDP-MurNAc-Ala (t_90_) (Figs. 2, 3). Also, UDP-MurNAc-Ala-Glu-Lys-Ala-Ala showed increased amount (*p* > 0.05) at t_90_, which is contrary to the alternations by treatment with cefotaxime only. Other noticeable effects of combination treatment were intensified glycolytic metabolic alterations compared with single usage. The amounts of 1,3-bP-glycerate and following metabolites of glycolysis until pyruvate were significantly increased, as well as amounts of phenylpyruvate, lactate, acetyl-CoA, and malonyl-CoA.

The changes in the intracellular amino acid metabolism caused by the treatment with cefotaxime were also found in the combination treatment with cefotaxime and azithromycin. Furthermore, all detected nucleotides with exception of GDP and GTP showed a decreased intracellular abundance, which was comparable to cefotaxime stress. Furthermore, similar effects were observed for adenine and uracil (Fig. 3).

In summary, cells stressed by combined treatment mostly showed metabolic changes similarly to cells stressed with cefotaxime (Fig. 3).

### DNA gyrase inhibitor moxifloxacin

The fluoroquinolone moxifloxacin had a significant effect on the amounts of 48 metabolites. The abundances of 29 metabolites were increased and 19 metabolites were decreased.

Most alterations were found for the purine and pyrimidine nucleotide metabolism. Levels of adenine, AMP, 2-dADP, 2-dATP, uracil, and the di- and triphosphates of uridine, cytidine, and guanosine were increased intracellularly. Reduced amounts of the other detected nucleotides were found after antibiotic treatment compared with control cells with exception of 2-dTMP, AMP, CMP, and UMP showing no changes (Fig. 3).

Intermediates of glycolysis such as 3-phosphoglycerate (t_30_, t_60_, and t_90_), 2-phosphoglycerate (t_30_, t_60_), PEP (t_90_), and pyruvate (t_15_) were increased. Pneumococci treated with moxifloxacin showed alterations in several amino acids like leucine, proline, glycine, serine, threonine, aspartate, glutamate, tryptophan, and more (*p* > 0.05) (Fig. 3). Changes of concentration of cell wall metabolites were also detected (Fig. 2). The amount of UDP-GlcNAc was increased significantly at t_15_–t_60_ and UDP-GlcNAc-enolpyruvate (t_90_) and GlcNAc-6-P (t_30_) were decreased. We found that pneumococci stressed with moxifloxacin contained enhanced concentrations of three building blocks of teichoic acids namely glycerol-1-phosphate, CDP-ribitol (both *p* < 0.05 at all time points), and CDP-choline (*p* > 0.05) (Fig. 3). As specific as in the case of the building blocks for teichoic acids, abundance of 4-P-pantothenate was increased in the cells at t_15_ and t_30_ after stress induction.

### Teixobactin-Arg10 stress metabolic alterations

The lipid II binder teixobactin-Arg10 caused alterations in concentrations of 65 pneumococcal metabolites compared with control. Amounts of 29 metabolites were increased and 36 metabolites decreased significantly. Teixobactin-Arg10 caused enrichment of cell wall precursors UDP-MurNAc, UDP-MurNAc-Ala-Glu-Lys, and UDP-MurNAc-Ala-Glu-Lys-Ala-Ala and also influenced concentrations of other intermediates of peptidoglycan synthesis such as GlcNAc-6P, UDP-GlcNAc, and UDP-GlcNAc-enolpyruvate (Fig. 2). Abundance of glycerol-1-phosphate was increased from t_15_ to t_60_ after stress induction.

**Fig. 4:**
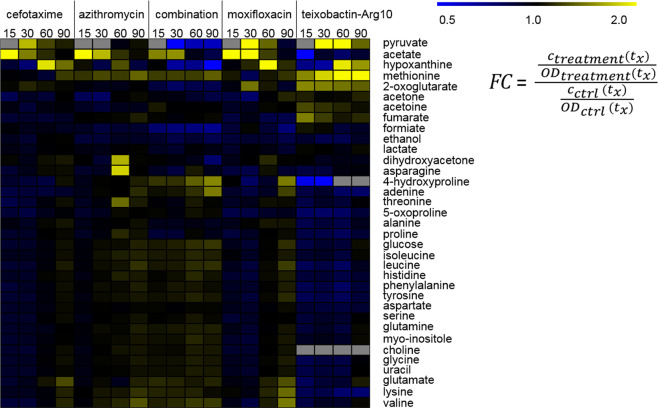
Extracellular metabolites of *S. pneumoniae* TIGR4*Δcps*. The illustrated heat map with fold changes (FC) of metabolite amounts after addition of antibiotics referred to control conditions both with normalization to optical density (600 nm) shows metabolites with higher concentrations compared to control in yellow and with lower concentrations in blue at the different time points (t_15_–t_90_). Gray fields: metabolites were not detected under stress and/or control conditions

The intracellular amounts of glycolysis intermediates and lactate were increased in pneumococci shortly after stress induction e.g., phosphoenolpyruvate (*p* > 0.05) and pyruvate at t_15_ and t_30_ after treatment and phenylpyruvate, as well as acetyl-CoA at t_15_. However, 90 min after stress induction the level of pyruvate was decreased compared to control conditions (Fig. 3). Increased concentrations were also detected for pyruvate extracellularly (Fig. 4). Another effect of the teixobactin analog was observed in the changed arginine-ornithine antiporter regulation. Ornithine secretion stopped after treatment with teixobactin-Arg10, but uptake of arginine was unchanged compared to control and other stress conditions (Fig. S4). In summary, teixobactin-Arg10 caused most metabolic changes in the exometabolome, for example observed for phenylalanine (t_15_ and t_60_), tyrosine (t_15_), serine (t_15_), asparagine (t_15_). Levels of amino acids like glycine, serine, tyrosine, proline, aspartate and asparagine were increased intracellularly shortly after beginning of stress (t_15_–t_30_). The same observations were made for glutamine and leucine (*p* > 0.05). Uptake or secretion of amino acids was not influenced. In contrast concentrations of most amino acids dropped down intracellularly under stress conditions compared to control at t_90_ (*p* > 0.05) (Figs. 3, 4).

Among the 36 metabolites with decreased amounts were many purine and pyrimidine metabolites, for instance all detected nucleotides and deoxy nucleotides showed lower concentrations at t_60_ and t_90_ after teixobactin-Arg10 stress when compared to the control. Also, lower abundance was found for 6-phosho gluconate. Only adenine (*p* < 0.05 for all time points) and uracil (*p* < 0.05 for t_15_–t_60_) level were increased intracellularly compared to control conditions (Fig. 3).

### General stress-dependent metabolic changes

The amounts of amino acids and cell wall precursors as well as nucleotides were altered in pneumococci by all stress conditions. The intracellular methionine amount was increased at t_15_ and t_30_. In contrast, other amino acid concentrations were reduced at t_90_ e.g., concentrations of lysine, isoleucine (both *p* < 0.05), glutamine and ornithine (both *p* > 0.05). The amounts of the nucleotides ADP, ADP-ribose, ATP, c-di-AMP, GMP, UMP, 2-dCTP, 2-dTDP and 2-dTTP decreased intracellularly during all stress conditions at t_90_ (Fig. 3). Furthermore, levels of ribose-5-P, malonyl-CoA and UDP-GlcNAc-enolpyruvate decreased intracellularly at t_90_ (Figs. 2, 3). A general increase in concentration was only observed for NADP^+^ at all time points with exception of treatment with azithromycin causing only increase in NADP^+^ level at t_90_ (Fig. 3).

## Discussion

The traditional therapies for pneumococcal-induced pneumonia include *β*-lactam, macrolide, fluoroquinolone and tetracycline antibiotics [36]. Resistance to penicillin and macrolide antibiotics in *S. pneumoniae* has increased in many areas [37]. Small molecules like antibiotics can have pleiotropic effects on bacteria, which was demonstrated in our study. Our investigations were focused on the non-encapsulated strain *S. pneumoniae* TIGR4*Δcps*. Specific differences in metabolism of *S. pneumoniae* strains have to be considered for a generalization of our results. We used antimicrobial compounds with classical and novel targets towards the bacterial metabolism at a single concentration to cover a broad range of responses in metabolic pathways. Including this study, several OMICs studies have been performed to demonstrate the effect of antibiotics on pneumococcal physiology [38–45].

### Impact of antibiotics as general metabolic changes

The AEC as ratio of ATP, ADP, and AMP describes the energy balance of cells. In living cells, the AEC is near 0.85, if the formation of ATP is controlled primarily by the concentration of the regulatory end product [46]. Chapman et al. showed that depletion of the carbon source glucose caused a decreased AEC in *Escherichia coli* [47]. In this study, during the whole experiment pneumococci had no limitation of glucose. At t_90_ after antibiotic treatment, about 22 mM glucose was available for the bacteria. The antibiotic treatment caused reduced levels of adenosine nucleotides but not affected the resulting AEC, so energy balance of the bacteria seems not to be influenced.

General, the effect of enhanced amino acid levels is described to be a stress response of *S. pneumoniae* to antibiotics [10, 48]. Bacterial stress mechanisms induce an increased synthesis of amino acids, which are precursors for protection proteins like CodY [49]. Methionine is essential for growth of pneumococcal cells [10, 50] and intracellular methionine concentration was increased immediately after initiation of stress conditions. Methionine is involved in many anabolic reactions like protein biosynthesis, *N*-formylmethionine formation and *S*-adenosylmethionine synthesis. Studies showed that a mutation of the MetQNP ABC-transporter and the methionine synthetase metQ/metEF induced an inhibition of pneumococcal growth [50, 51]. *S. pneumoniae* might show an adaptation in the metabolism in raising the level of methionine to protect the cells.

The intracellular level of c-di-AMP significantly decreased in all stressed cells at t_90_ after addition of antibiotics. For *Staphylococcus aureus* a very low level of c-di-AMP is supposed to contribute to reduced bacterial growth rate [52]. Cell wall biosynthesis seems to be affected not only by specific inhibitors like cefotaxime because diverse effects on synthesis of cell wall precursors were observed for all antibiotics. However, all stress conditions led to significant reduction in levels of the cell wall precursor UDP-GlcNAc-enolpyruvate. This might be connected with a general influence of antibiotic treatment on glutamine metabolism of *S. pneumoniae* resulting in decreased level of glutamine and Glc-NAc-6-P at t_90_.

Another general effect seems to be a downregulation of PPP providing reduction equivalents by reducing NADP^+^. NADP^+^ was detected in increased amounts and ribose-5-phosphate, as another important product of PPP, in decreased amounts after all stress conditions. Lower level of ribose-5-phosphate as precursor of nucleotide biosynthesis might be connected with dropping of nucleotide amounts at t_90_. Increased amounts of NADP^+^ were shown in *S. aureus* among different antibiotic treatments only with fluoroquinolone ciprofloxacin [53].

### Impact of cephalosporin cefotaxime on the metabolism of *S. pneumoniae*

Metabolic response of *S. pneumoniae* to cefotaxime showed an increased amount of glutamine and glutamate intracellularly. Glutamine is major nitrogen donor for purine and pyrimidine biosynthesis and for the synthesis of the cell wall precursors. Glutamine is used by the aminotransferase GlmS to convert fructose-6-P into glucosamine-6-P [54, 55]. As shown in *S. aureus* inhibition of GlmS correlates with a broad set of cell wall synthesis inhibitors [56]. Transcriptional response of *S. pneumoniae* to penicillin showed decreased glutamine metabolism. The most downregulated genes encode ABC transporter GlnQ, the transcriptional regulator GlnR, and the glutamine synthetase GlnA [38]. Enhanced intracellular concentrations of glutamine and glutamate as described for *S. pneumoniae* after penicillin treatment [38] were also observed after exposure to cefotaxime in this study whereas uptake of glutamine and glutamate was not influenced. Interestingly, it was shown that glutamine protects *S. pneumoniae* against penicillin stress [38]. The inhibition of GlmS in methicillin-resistant *S. aureus* [56] and of GlnA in penicillin-resistant *S. pneumoniae* [38] decreased their level of resistance. Glutamine is also a cofactor for the cross-linking of the peptidoglycan by MurT/GatD [57]. Our results showed that cefotaxime influenced the amounts of four peptidoglycan precursors reaffirming the known mechanism of action of cephalosporins [58].

Increased amounts of threonine were detected in pneumococcal stress response. Threonine is part of the active site of penicillin binding protein 2 × (PBP2x). After binding of cefotaxime to PBP2x, threonine has directly contact with the cephalosporin [59]. Loss of the hydrogen bond between cefotaxime and threonine is known to induce resistance [60]. Thus, threonine could be a crucial metabolite in formation of resistance. In this study, cefotaxime stress increased the amount of threonine intracellularly.

The results of the metabolome analysis revealed variations of peptidoglycan biosynthesis. Therefore, the pneumococcal cell morphology was investigated and visualized. The cell wall was not affected in thickness or morphology. Conspicuously were the white areas in the TEM images. Hoyer et al. could show similar effects depending on medium composition that also strongly influenced the protein expression in *S. pneumoniae* [61]. The areas in the TEM images could be granular of a storage substance that was not detectable by metabolome analysis. Poly-β-hydroxybutyrate (PHB) is an intracellular storage material found in *Lactobacillus, Lactococcus, Pediococcus,* and *Streptococcus* species. The bacteria accumulate PHB during the stationary phase of growth [62]. However, for *S. pneumoniae* PHB is hitherto unknown.

### Impact of macrolide azithromycin on the metabolism of *S. pneumoniae*

Macrolides have shown some strong anti-inflammatory effects [63], reducing the release of IL-8 and TNF-α. Also macrolides inhibit effective adherence of bacteria to respiratory epithelial cells and so decrease the production of virulence factors [64]. It has been demonstrated that azithromycin blocks peptide biosynthesis resulting in a reduced uptake of amino acids from the medium [53]. Our findings showed no significant changes of amino acid uptake and intracellular concentrations by azithromycin stress. Interestingly, macrolides can be secreted by ATP dependent efflux pumps from pneumococcal cells [65, 66]. A study using *E. coli* has suggested a physical interaction between macrolide efflux proteins Mef(E) and Mel, binding of macrolides to Mel and localization to the membrane [67]. Induction of efflux proteins occurs very fast after exposure to different macrolide antibiotics [68]. Efflux of antibiotics could be a reason that *S. pneumoniae* showed least metabolic alteration caused by azithromycin compared with other stress conditions. In this study, azithromycin treatment of *S. pneumoniae* influenced the purine and pyrimidine biosynthesis as also proved for *S. aureus* [53, 69]. These results suggested that the bacterial cells responded to azithromycin by reducing generation of nucleotides and resources for DNA synthesis [69].

### Impact of combination of cefotaxime and azithromycin on the metabolism of *S. pneumoniae*

A drug combination therapy is increasingly used in the hospitals because of better coverage in polymicrobial CAP. It has been suggested that *S. pneumoniae*-induced pneumonia patients have concomitant *Mycoplasma pneumoniae* or *Legionella sp*. infections [6, 63]. Combination therapy acts at two different sites in bacteria i.e., the inhibition of cell wall biosynthesis by *β*-lactams, and inhibition of protein synthesis by macrolides. Our study showed for the first time metabolome adaptations of *S. pneumoniae* to combined stress of cefotaxime and azithromycin. Combined application of these two antibiotics resulted in a largest change in the metabolome of *S. pneumoniae* compared to the other antimicrobial treatments. Comparable metabolic changes were observed when cefotaxime was applied solely, e.g., on amino acid metabolism. But combination treatment seemed to intensify the effects on glycolysis and lactate formation as well as nucleotide biosynthesis compared to cefotaxime alone. However, there were also significant changes of seven metabolites only caused by the combined treatment with cefotaxime and azithromycin. Probably these additional effects on the pneumococcal metabolism could be a reason for better outcome of CAP patients. Glutathione is used as a marker of oxidative stress [70]. Only after treatment with cefotaxime and azithromycin an altered level of oxidized glutathione (GSSG) was found.

### Impact of fluoroquinolone moxifloxacin on the metabolism of *S. pneumoniae*

The treatment of *S. pneumoniae* with moxifloxacin causes double-stranded breaks in the bacterial chromosome [42] and requires active protein synthesis [71]. Treatment with moxifloxacin was reported not altering the level of global supercoiling [42]. Moxifloxacin induces transcriptional changes, which ultimately stimulate the Fenton reaction, increasing ROS accumulation and contributing to cell death [42]. *S. pneumoniae* treated with the fluoroquinolone induces upregulation of the *fatDCEB* operon coding an iron transporter. Subsequently, the intracellular iron concentration increased leading to accumulation of ROS [72]. De la Campa et al. reviewed that for the facultative anaerobic bacterium, *S. pneumoniae*, the increased lethality of fluoroquinolones is mediated by an increase in ROS fiting with the antibiotic lethality model proposed for aerobic bacteria [40].

The transcriptomic response of *S. pneumoniae* R6 to moxifloxacin led to an upregulation of metabolic pathways involved in the production of pyruvate [42]. Similar to Ferrandiz et al. which showed an increase in acetyl-CoA [42], our study demonstrated also increased abundances of glycolytic intermediates and pyruvate. Interestingly, acetyl-CoA showed no altered level compared to the control in this study but increase in amount of acetate extracellularly. The inhibition of DNA and RNA synthesis led to a block in septum formation and a more thickened cell wall [73]. Morphological changes of cell wall could be confirmed in our study. In *S. aureus*, a downregulation of metabolism on transcriptomic level was found by treatment with the fluoroquinolone ciprofloxacin, including purine and pyrimidine biosynthesis [69]. Furthermore, in *S. aureus* an increase of nucleotides and nucleosides was detected [53]. Also genes of purine and pyrimidine biosynthesis were upregulated in *S*. *pneumoniae* R6 treated with moxifloxacin [42]. Both fits well with our results, nearly half of all detected nucleotides were increased in amounts significantly in *S. pneumoniae* cells under moxifloxacin stress. Changed levels of cell wall intermediates as observed for *Streptococcus faecalis* [73] were also detected in our study. CDP-ribitol was significantly influenced only by moxifloxacin treatment, so it could function as a marker of moxifloxacin stress in our study. Increased amounts of CTP might be connected with the enhanced level of CDP-ribitol. Further investigations of *S. pneumoniae* on metabolome level are necessary including all other antibiotic drug classes to confirm this metabolite as marker.

### Impact of Arg10 teixobactin on the metabolism of *S. pneumoniae*

The need for new antibiotics for treatment of resistant pathogens become a major global concern for human health. The isolation of teixobactin in 2015 by Ling et al. raised great expectations [74]. It was one of a few novel antibiotics that have been reported in recent years showing no resistance development [28]. The naturally occurring molecule, teixobactin, has the same limitations due to the low yield [29]. Pamar et al. designed and synthesized potent teixobactin analogs [31, 75]. This was the first study that investigated the effects of a teixobactin analog on bacterial metabolism by using teixobactin-Arg10. Lipid II as one of the known targets of teixobactin is removed from the cytoplasmic membrane prior to incorporation of its disaccharide-peptide moiety into peptidoglycan [76]. The binding of teixobactin prevents the removal of lipid II. The pyrophosphate group and the attached first sugar subunits of lipid II was found to be a minimal motif for stabile teixobactin binding [77]. In our study, we observed an accumulation of the three peptidoglycan precursors UDP-MurNAc, UDP-MurNAc-Ala, and UDP-MurNAc-Ala-Glu-Lys-Ala-Ala confirming the inhibition of peptidoglycan synthesis in *S. pneumoniae* treated with teixobactin-Arg10. Formation of excrescence found in transmission electron micrographs of *S. pneumoniae* TIGR4*Δcps* cells pointed out changes in cell wall morphology. Teixobactin has an effect on cell morphology and destroyed cell wall integrity in *S. aureus* [78]. Our observations confirmed these results for *S. pneumoniae* TIGR4Δ*cps* treated with the teixobaction analog.

The main metabolic pathway of *S. pneumoniae* is glycolysis [48]. Therefore, influences on glycolysis should be significant and changes are expected in metabolome. It is possible that increase of glycolysis intermediates indicates a stress response in this study. However, 90 min after antimicrobial stress, glycolysis metabolites are present in a decreased concentration compared with unstressed cells. Possibly the metabolism is downregulated by stress and consequently less energy is available.

Another interesting outcome was that the extracellular level of ornithine was influenced by the stress induced by teixobactin-Arg10. Arginine-ornithine antiporter (ArcD) is essential for the virulence of *S. pneumoniae* TIGR4 [79]. Arginine is one of the amino acids that cannot be synthesized *de novo* and has to be taken up by pneumococcus [9, 48]. Uptake of arginine was unaffected but ornithine secretion was completely stopped by treatment with the teixobactin analog. Gupta et al. assumed another function for ArcD that affects the linkage of the capsular polysaccharide to pneumococcal cell wall [80], however, this aspect is not relevant for the non-encapsulated *S. pneumoniae* TIGR4*Δcps*. The deletion of *arcD* reduces the amount of capsule materials on the bacterial surface. ArcD appears to play different roles in the pathogenicity of various pneumococcal serotypes, as the deficiency of ArcD in another strain does not affect the capsule [80, 81].

Pyrimidine nucleotides play an important role in the biosynthesis of activated nucleotide sugars (NDP-sugars). NDP-sugars are precursors for the synthesis of exopolysaccharides and peptidoglycan in lactic acid bacteria [82]. Carvalho et al. proposed a link between pyrimidine metabolism and capsule biosynthesis in *S. pneumoniae* D39 [83, 84]. Also they showed that uracil affects the pneumococcal capsule [85]. In our study, uracil concentration intracellularly increased after antibiotic stresses (teixobactin-Arg10, moxifloxacin, cefotaxime, and combination of cefotaxime and azithromycin) and adenine concentration showed same changes. Cultivation medium was supplemented with both nucleobases, but uptake was not enhanced compared with control. Due to the use of non-encapsulated *S. pneumoniae* TIGR4, in our study uracil was not associated with capsule formation. High amounts of nucleobases and decreased level of nucleotides might refer to intensified degradation of nucleotides or inhibition of salvage pathways of nucleotide formation as an outcome of reduced growth.

## Conclusion and outlook

The presented study showed intra- and extracellular metabolic changes of *S. pneumoniae* TIGR4*Δcps* stressed by different antimicrobial compounds. Possibly there are limitations in this approach resulting from the usage of only a single strain of *S. pneumoniae* and antibiotic concentration. Based on our findings, future investigations on metabolic adaptions of *S. pneumoniae* to antibiotic treatment should include different *S. pneumoniae* strains to exclude strain-specific reactions. The use of resistant bacteria utilizing resistance to a specific antimicrobial compound or compound class may allow to verify metabolic alterations to antibiotic stress in nonresistant strains. Furthermore, challenging bacteria with different concentrations of antibiotics could be helpful to study dose-dependent changes in metabolism.

No antibiotic showed a single alteration of a specific metabolite or a specific metabolic pathway in pneumococcal cells but rather a globally affected metabolome. Distinctive pathways affected were the purine and pyrimidine metabolism and the peptidoglycan biosynthesis as well. The fluoroquinolone moxifloxacin altered the nucleotide metabolism and teichoic acid precursors in a specific way. Typical for treatment with cefotaxime were enhanced levels of glutamine and glutamate and altered peptidoglycan metabolism. The fewest changes in the metabolome were caused by treatment with azithromycin, only remarkable changes in nucleotide metabolism were observed. A combination of cefotaxime and azithromycin resulted in metabolic variations mostly comparable to the application of cefotaxime alone. The teixobactin-Arg10 influenced level of peptidoglycan precursors as well as nucleotides and the arginine–ornithine antiporter. Given the broader global effects of the synthetic teixobactin analog on the metabolome of pneumococcal cells, it potentially provides a starting point for a new antibiotic treatment strategy.

## Supplementary information

Supplemental Tables Leonard et al.

Supplemental Figures Leonard et al.
