# Supplementary material for: Exploring metabolic adaptation of Streptococcus pneumoniae to antibiotics
Source: J Antibiot (Tokyo). 2020 Mar 24;73(7):441–54. doi: 10.1038/s41429-020-0296-3 (PMC7292801; doi:10.1038/s41429-020-0296-3)
Supplement: Supplementary file 1 — Supplemental Tables Leonard et al. [file 41429_2020_296_MOESM1_ESM.pdf]

Supplemental material

Exploring metabolic adaptation of Streptococcus pneumoniae to antibiotics

Anne Leonard[a], Kevin Möhlis[a], Rabea Schlüter[b], Edward Taylor[c], Michael Lalk[a], Karen Methling[a*]

[a] University of Greifswald, Institute for Biochemistry, Metabolomics, Felix-Hausdorff-Str. 4, 17489 Greifswald, Germany

[b] University of Greifswald, Imaging Center of the Department of Biology, F.-L-Jahn-Str. 15, 17489 Greifswald, Germany

[c] University of Lincoln, School of Life Sciences, Green Lane, LN67DL, Lincoln, England

[*]Corresponding author. Tel.: + 49 (0)3834 420-4167; fax: + 49 (0)3834 420-4479.
E-mail address: methling@uni-greifswald.de (K. Methling)

Keywords: *Streptococcus pneumoniae*, metabolism, antibiotics, antimicrobial substance, adaptation

Supplementary tables

Table of contents:

TableS.1: Internal standards for GC-MS and HPLC-MS analysis.

TableS.2: Summary of identified metabolites with internal standard used for quantification.

TableS.3: Fold changes of extracellular metabolites at all sampling time points.

TableS.4: Fold changes of intracellular metabolites at all sampling time points.

TableS.5: Metabolites measured by GC-MS and identified by database alignment.

TableS.6: Calculated and detected masses of metabolites measured by HPLC-MS and identified by database alignment (Human Metabolome Database).

TabS.1: Internal standards for HPLC-MS and GC-MS analysis.

Metabolite	labeled	[nmol/sample]	Company	^{13}C	D	^{15}N
**HPLC-MS**						
AMP	$^{13}C_{10}{}^{15}N_5$	4	SIGMA-ALDRICH			
ATP	$^{13}C_{10}{}^{15}N_5$	35	Silantes GmbH			
CMP	$^{13}C_9{}^{15}N_3$	3	SIGMA-ALDRICH			
CTP	$^{13}C_9{}^{15}N_3$	7	Silantes GmbH			
fructose-1,6-bisphosphate	$^{13}C_6$	40	Cambridge Isotope Laboratories, Inc.			
GTP	$^{13}C_{10}{}^{15}N_5$	15	Silantes GmbH			
UMP	$^{13}C_9{}^{15}N_2$	3	SIGMA-ALDRICH			
UTP	$^{13}C_9{}^{15}N_2$	25	Silantes GmbH			
**GC-MS**				^{13}C	D	^{15}N
alanine	$^{13}C^{15}ND$	82	Cambridge Isotope Laboratories, Inc.	3	4	1
citrate	$^{13}C_6$	40	SIGMA-ALDRICH			
fumarate	$^{13}C_4$	40	SIGMA-ALDRICH			
glucose	$^{13}C_6$	40	SIGMA-ALDRICH			
L-arginine	$^{13}C^{15}ND$	32	Cambridge Isotope Laboratories, Inc.	6	7	4
L-asparagine	$^{13}C^{15}ND$	45	Cambridge Isotope Laboratories, Inc.	4	3	2
L-aspartate	$^{13}C^{15}ND$	39	Cambridge Isotope Laboratories, Inc.	4	3	1
L-cystine	$^{13}C^{15}ND$	2	Cambridge Isotope Laboratories, Inc.	6	6	2
L-glutamate	$^{13}C^{15}ND$	66	Cambridge Isotope Laboratories, Inc.	5	5	1
L-glutamine	$^{13}C^{15}ND$	45	Cambridge Isotope Laboratories, Inc.	5	5	2
glycine	$^{13}C^{15}ND$	44	Cambridge Isotope Laboratories, Inc.	2	2	1
L-histidine	$^{13}C^{15}ND$	1	Cambridge Isotope Laboratories, Inc.	6	5	3
L-isoleucine	$^{13}C^{15}ND$	45	Cambridge Isotope Laboratories, Inc.	6	10	1
L-leucine	$^{13}C^{15}ND$	76	Cambridge Isotope Laboratories, Inc.	6	10	1
L-lysine	$^{13}C^{15}ND$	67	Cambridge Isotope Laboratories, Inc.	6	9	2
L-malate	$^{13}C_3$	40	Cambridge Isotope Laboratories, Inc.			
L-methionine	$^{13}C^{15}ND$	39	Cambridge Isotope Laboratories, Inc.	5	8	1
L-phenylalanine	$^{13}C^{15}ND$	31	Cambridge Isotope Laboratories, Inc.	9	8	1
L-proline	$^{13}C^{15}ND$	47	Cambridge Isotope Laboratories, Inc.	5	7	1
L-serine	$^{13}C^{15}ND$	37	Cambridge Isotope Laboratories, Inc.	3	3	1
L-threonine	$^{13}C^{15}ND$	41	Cambridge Isotope Laboratories, Inc.	4	5	1
L-tryptophan	$^{13}C^{15}ND$	48	Cambridge Isotope Laboratories, Inc.	11	8	2
L-tyrosine	$^{13}C^{15}ND$	44	Cambridge Isotope Laboratories, Inc.	9	7	1
L-valine	$^{13}C^{15}ND$	53	Cambridge Isotope Laboratories, Inc.	5	8	1
N,N- dimethylphenylalanine	-	40	SIGMA-ALDRICH			
L-ornithine	$^{13}C_5$	40	SIGMA-ALDRICH			
p-chlorophenylalanine	-	40	Bachem			
pyruvate	$^{13}C_3$	40	SIGMA-ALDRICH			
ribitiol	-	40	Merck			
succinate	$^{13}C_4$	40	SIGMA-ALDRICH			

TabS.2: Summary of identified metabolites with internal standard used for quantification.
Labeling of amino acid internal standards as mentioned in TabS.1.

**Metabolite**	**labeled internal standard**	
**GC-MS**		
2-oxoglutarate	*N,N*-dimethylphenylalanine	
2-phosphoglycerate	*N,N*-dimethylphenylalanine	
3-hydroxybutyrate	*N,N*-dimethylphenylalanine	
3-phosphoglycerate	N,N-dimethylphenylalanine	
4-hydroxyproline	L-proline	$^{13}C^{15}ND$
5-oxoproline	L-proline	$^{13}C^{15}ND$
alanine	alanine	$^{13}C^{15}ND$
asparagine	L-asparagine	$^{13}C^{15}ND$
aspartate	L-aspartate	$^{13}C^{15}ND$
cysteine	cysteine	$^{13}C^{15}ND$
cystine	L-cystine	$^{13}C^{15}ND$
dihydroxyacetone phosphate	*N,N*-dimethylphenylalanine	
fructose-6-phosphate	glucose	$^{13}C_6$
fructose	glucose	$^{13}C_6$
fumarate	fumarate	$^{13}C_4$
glucose-6-phosphate	glucose	$^{13}C_6$
glucose	glucose	$^{13}C_6$
glucuronate	glucose	$^{13}C_6$
glutamate	L-glutamate	$^{13}C^{15}ND$
glutamine	L-glutamate	$^{13}C^{15}ND$
glycine	L-glycine	$^{13}C^{15}ND$
histidine	*N,N*-dimethylphenylalanine	
lactate	pyruvate	$^{13}C_3$
leucine	L-leucine	$^{13}C^{15}ND$
lysine	L-lysine	$^{13}C^{15}ND$
methionine	L-methionine	$^{13}C^{15}ND$
myo-inositol	*N,N*-dimethylphenylalanine	
ornithine	L-ornithine	$^{13}C_5$
phenylalanine	*N,N*-dimethylphenylalanine	
phenylpyruvate	pyruvate	$^{13}C_3$
phosphoenolpyruvate	pyruvate	$^{13}C_3$
proline	L-proline	$^{13}C^{15}ND$
pyruvate	pyruvate	$^{13}C_3$
serine	L-serine	$^{13}C^{15}ND$
succinate	succinate	$^{13}C_4$
threonine	L-threonine	$^{13}C^{15}ND$
tryptophan	L-tryptophan	$^{13}C^{15}ND$
tyrosine	L-tyrosine	$^{13}C^{15}ND$
valine	L-valine	$^{13}C^{15}ND$

Metabolite	labeled internal standard	
**HPLC-MS**		
1,3-bisphosphoglycerate	ATP	$^{13}C_{10}{}^{15}N_5$
2-dADP	AMP	$^{13}C_{10}{}^{15}N_5$
2-dATP	ATP	$^{13}C_{10}{}^{15}N_5$
2-dCTP	CTP	$^{13}C_9{}^{15}N_3$
2-dTDP	AMP	$^{13}C_{10}{}^{15}N_5$
2-dTMP	AMP	$^{13}C_{10}{}^{15}N_5$
2-dTTP	UTP	$^{13}C_9{}^{15}N_2$
6-phospho gluconate	AMP	$^{13}C_{10}{}^{15}N_5$
acetyl-CoA	ATP	$^{13}C_{10}{}^{15}N_5$
ADP	AMP	$^{13}C_{10}{}^{15}N_5$
ADP-glucose	ATP	$^{13}C_{10}{}^{15}N_5$
ADP-ribose	ATP	$^{13}C_{10}{}^{15}N_5$
AMP	AMP	$^{13}C_{10}{}^{15}N_5$
ATP	ATP	$^{13}C_{10}{}^{15}N_5$
c-di-AMP	AMP	$^{13}C_{10}{}^{15}N_5$
CDP	AMP	$^{13}C_{10}{}^{15}N_5$
CDP-choline	AMP	$^{13}C_{10}{}^{15}N_5$
CDP-ribitol	AMP	$^{13}C_{10}{}^{15}N_5$
CMP	AMP	$^{13}C_{10}{}^{15}N_5$
CTP	CTP	$^{13}C_9{}^{15}N_3$
FAD	ATP	$^{13}C_{10}{}^{15}N_5$
FMN	ATP	$^{13}C_{10}{}^{15}N_5$
fructose-1,6-bisphosphate	fructose-1,6-bisphosphate	$^{13}C_6$
GDP	AMP	$^{13}C_{10}{}^{15}N_5$
GlcNAc-/GalNAc-/ManNAc-6P	fructose-1,6-bisphosphate	$^{13}C_6$
GMP	UMP	$^{13}C_9{}^{15}N_2$
GSSG	UMP	$^{13}C_9{}^{15}N_2$
GTP	GTP	$^{13}C_{10}{}^{15}N_5$
IMP	AMP	$^{13}C_{10}{}^{15}N_5$
L-myo-inositol triphosphate	ATP	$^{13}C_{10}{}^{15}N_5$
malonyl-CoA	ATP	$^{13}C_{10}{}^{15}N_5$
NAD^+	UMP	$^{13}C_9{}^{15}N_2$
$NADP^+$	ATP	$^{13}C_{10}{}^{15}N_5$
pantothenate	AMP	$^{13}C_{10}{}^{15}N_5$
pantothenate-4-phosphate	AMP	$^{13}C_{10}{}^{15}N_5$
PRPP	UTP	$^{13}C_9{}^{15}N_2$
ribose-5-phosphate	UMP	$^{13}C_9{}^{15}N_2$
sedoheptulose-1,7-bisphosphate	fructose-1,6-bisphosphate	$^{13}C_6$
sedoheptulose-7-phosphate	fructose-1,6-bisphosphate	$^{13}C_6$
UDP	AMP	$^{13}C_{10}{}^{15}N_5$
UDP-GlcNAc/-GalNAc	UTP	$^{13}C_9{}^{15}N_2$
UDP-GlcNAc-enolpyruvate	UTP	$^{13}C_9{}^{15}N_2$
UDP-glucose/galactose	UTP	$^{13}C_9{}^{15}N_2$
UDP-MurNAc	UTP	$^{13}C_9{}^{15}N_2$
UDP-MurNAc-Ala	UTP	$^{13}C_9{}^{15}N_2$
UDP-MurNAc-Ala-Glu-Lys	AMP	$^{13}C_{10}{}^{15}N_5$
UDP-MurNAc-Ala-Glu-Lys-Ala-Ala	AMP	$^{13}C_{10}{}^{15}N_5$
UMP	UMP	$^{13}C_9{}^{15}N_2$
UTP	UTP	$^{13}C_9{}^{15}N_2$
XMP	fructose-1,6-bisphosphate	$^{13}C_6$

TabS.3: Fold changes of extracellular metabolite concentrations [mM] at every sampling time point t_x were calculated and normalized to the optical density of the bacterial suspension at the respective sampling time. Mean values are listed.

mean value $[(c_{treatment}(t_x)/OD_{treatment}(t_x))/(c_{control}(t_x)/OD_{control}(t_x))]$	cefotaxime				azithromycin				combination of cefotaxime and azithromycin			
	t_{15}	t_{30}	t_{60}	t_{90}	t_{15}	t_{30}	t_{60}	t_{90}	t_{15}	t_{30}	t_{60}	t_{90}
2-oxoglutarate	1.030	1.144	1.076	1.029	0.976	0.952	0.954	0.994	0.880	0.809	0.853	0.844
4-hydroxyproline	0.902	0.951	1.042	1.050	0.919	0.939	1.842	1.007	0.937	0.876	1.105	0.946
5-oxoproline	0.895	0.872	0.947	0.986	0.916	0.944	1.042	1.008	1.020	0.974	1.070	1.035
acetate	2.222	1.508	1.172	1.001	2.443	1.739	1.195	1.043	1.526	1.583	0.954	0.855
acetoine	0.967	0.891	0.963	0.982	0.931	0.892	0.947	0.949	1.101	0.994	0.992	0.996
acetone	0.843	0.928	0.893	1.002	0.834	0.808	0.990	1.040	0.975	0.965	0.912	0.868
adenine	0.871	0.891	0.887	0.910	0.950	1.020	1.005	1.171	1.057	1.083	1.190	1.493
alanine	0.972	0.936	1.029	1.081	0.955	0.986	1.009	1.062	0.951	0.948	0.967	0.941
arginine	0.837	0.846	0.887	0.970	0.866	0.896	0.909	0.961	1.030	0.945	1.058	1.021
asparagine	0.912	0.891	0.876	1.065	0.950	1.012	1.007	1.118	1.212	1.196	1.385	1.538
aspartate	0.921	0.937	0.967	1.041	0.978	1.011	0.986	1.023	1.020	0.991	1.024	1.023
choline	0.917	0.906	0.983	1.048	0.958	1.010	1.076	1.081	1.043	1.036	1.108	1.098
dihydroxyacetone	1.027	0.956	1.059	1.094	1.006	0.904	1.709	1.017	1.008	0.974	0.954	0.785
ethanol	0.942	0.907	0.996	1.013	0.888	0.893	0.933	0.927	0.911	0.859	0.903	0.843
formate	0.981	0.948	0.963	0.935	0.934	0.879	0.909	0.910	0.787	0.763	0.754	0.724
fumarate	0.899	0.918	0.903	0.929	1.006	1.014	1.028	1.117	1.019	0.979	1.009	1.033
glucose	0.912	0.925	0.975	1.065	0.973	1.046	1.048	1.131	1.101	1.122	1.184	1.214
glutamate	0.912	0.950	1.081	1.297	0.967	1.038	0.944	1.130	1.163	1.138	1.097	1.145
glutamine	0.919	0.934	0.956	1.007	0.982	1.045	1.029	1.111	1.075	1.094	1.121	1.118
glycine	0.920	0.929	0.989	1.059	0.971	1.027	1.039	1.095	1.065	1.070	1.111	1.087
histidine	0.933	0.924	1.017	1.049	0.964	1.023	1.120	1.100	1.019	1.056	1.157	1.076
hypoxanthine	0.902	0.784	1.904	1.447	1.056	0.861	1.320	0.875	1.006	0.698	0.729	0.421
isoleucine	0.914	0.924	0.973	1.085	0.998	1.060	1.114	1.234	1.096	1.115	1.211	1.241
lactate	0.964	0.939	0.992	1.002	0.965	0.956	0.990	1.015	0.935	0.912	0.987	0.957
leucine	0.905	0.914	0.980	1.102	0.980	1.053	1.077	1.197	1.090	1.099	1.175	1.188
lysine	0.939	0.942	0.977	1.136	1.003	1.083	1.085	1.202	1.143	1.133	1.135	1.047
methionine	0.989	0.975	0.903	1.234	1.207	1.288	1.238	1.403	1.242	1.252	1.247	1.378
myo-inositole	0.912	0.931	0.975	1.010	0.974	1.025	1.044	1.082	1.064	1.062	1.152	1.096
ornithine	1.084	1.066	1.112	1.140	1.000	1.059	1.029	1.079	0.940	1.068	0.977	0.989
phenylalanine	0.893	0.927	0.973	1.056	0.987	1.053	1.042	1.117	1.037	1.094	1.087	1.049
proline	0.914	0.925	1.006	1.037	0.930	0.973	1.161	1.070	0.895	0.902	1.025	0.951
pyruvate	1.514	1.738	1.277	1.118	0.134	0.859	0.966	1.066	0.125	0.186	0.642	0.631
serine	0.913	0.919	0.991	1.043	0.958	1.002	1.030	1.072	1.009	1.028	1.087	1.034
threonine	0.861	0.902	0.976	1.062	0.937	0.974	1.432	1.107	0.989	0.964	1.101	1.019
tyrosine	0.906	0.915	0.973	1.051	0.968	1.047	1.063	1.093	1.065	1.081	1.135	1.091
uracil	0.918	0.921	0.978	1.030	0.963	1.010	1.016	1.064	1.062	1.061	1.113	1.102
valine	0.890	0.892	0.974	1.151	0.983	1.095	1.154	1.334	1.133	1.166	1.236	1.232

mean value [$(c_{treatment}(t_x)/OD_{treatment}(t_x))/(c_{control}(t_x)/OD_{control}(t_x))$]	moxifloxacin				teixobactin-Arg10			
	t_{15}	t_{30}	t_{60}	t_{90}	t_{15}	t_{30}	t_{60}	t_{90}
2-oxoglutarate	0.971	1.460	1.094	0.915	1.545	1.521	1.463	1.381
4-hydroxyproline	0.879	0.858	1.064	0.911	0.872	0.882	0.968	0.977
5-oxoproline	0.860	0.870	0.921	0.943	0.804	0.802	0.806	0.890
acetate	2.580	2.502	1.249	0.946	0.498	0.914	0.804	0.902
acetoine	0.926	0.944	1.028	1.002	1.294	1.199	1.143	1.055
acetone	0.874	0.793	0.990	1.145	1.129	1.024	1.036	1.143
adenine	0.918	0.875	0.930	1.266	0.868	0.915	0.807	0.799
alanine	0.952	1.014	1.088	1.082	0.851	0.888	0.858	0.943
arginine	0.814	0.763	0.827	0.912	1.029	1.003	1.096	1.287
asparagine	0.993	0.805	0.919	1.493	0.542	0.294	0.016	0.002
aspartate	0.946	0.918	0.984	1.046	0.900	0.887	0.880	0.902
choline	0.913	0.914	1.006	1.074	0.357	0.377	0.407	0.487
dihydroxyacetone	0.950	0.998	1.163	1.015	1.003	0.942	0.958	0.952
ethanol	0.888	0.907	0.905	0.862	0.913	0.854	0.847	0.923
formate	0.891	1.027	0.896	0.767	1.076	1.035	0.890	0.924
fumarate	0.881	0.959	0.866	0.857	1.509	1.245	1.092	1.161
glucose	0.948	0.902	1.012	1.181	0.866	0.908	0.892	1.055
glutamate	0.972	0.952	1.127	1.320	0.826	0.896	0.906	1.107
glutamine	0.956	0.909	0.991	1.079	0.888	0.928	0.886	1.038
glycine	0.942	0.922	1.022	1.108	0.845	0.880	0.865	0.986
histidine	0.908	0.889	1.042	1.125	0.887	0.903	0.865	0.909
hypoxanthine	0.974	1.151	2.588	1.173	0.738	0.689	1.931	1.610
isoleucine	0.947	0.899	1.029	1.236	0.892	0.927	0.920	1.094
lactate	0.918	0.992	0.963	0.908	1.025	1.002	0.962	1.041
leucine	0.942	0.885	1.036	1.241	0.835	0.867	0.816	0.941
lysine	1.023	0.968	1.148	1.427	0.838	0.857	0.787	0.782
methionine	1.097	0.834	1.092	1.204	1.549	1.853	2.001	2.707
myo-inositole	0.921	0.896	1.012	1.092	0.932	0.957	0.924	1.036
ornithine	1.055	1.057	1.095	1.086	0.298	0.314	0.266	0.311
phenylalanine	0.933	0.921	1.013	1.116	0.843	0.873	0.811	0.887
proline	0.885	0.931	1.006	1.046	0.863	0.862	0.826	0.896
pyruvate	2.153	4.142	1.410	0.904	10.978	6.677	2.114	1.409
serine	0.898	0.929	1.000	1.009	0.890	0.922	0.906	1.005
threonine	0.927	0.952	1.059	1.044	0.862	0.922	0.892	0.990
tyrosine	0.939	0.890	1.035	1.168	0.835	0.861	0.831	0.890
uracil	0.940	0.915	0.998	1.074	0.860	0.889	0.873	0.999
valine	0.955	0.873	1.113	1.489	0.869	0.932	0.921	1.038

TabS.4: Fold changes of intracellular metabolite concentrations at every sampling time point t_x.

$FC = \frac{mean\ value\ t_x\ (treatment)}{mean\ value\ t_x (control)}$	cefotaxime				azithromycin				combination of cefotaxime and azithromycin			
	t_{15}	t_{30}	t_{60}	t_{90}	t_{15}	t_{30}	t_{60}	t_{90}	t_{15}	t_{30}	t_{60}	t_{90}
1,3-bisphosphoglycerate	1.532	1.056	0.857	1.121	1.113	0.803	0.748	1.130	1.745	1.260	0.931	1.251
2-dADP	0.978	1.022	0.776	0.540	0.895	0.865	0.814	0.672	0.723	0.663	0.582	0.404
2-dATP	1.000	1.041	0.769	0.577	0.936	0.918	0.794	0.642	0.834	0.789	0.626	0.455
2-dCTP	0.865	0.833	0.692	0.457	0.760	0.723	0.756	0.500	0.801	0.789	0.609	0.333
2-dTDP	0.613	0.584	0.532	0.295	0.800	0.664	0.808	0.354	0.514	0.438	0.362	0.206
2-dTMP	1.017	0.994	0.761	0.498	0.751	0.767	0.869	0.668	1.095	0.950	0.831	0.547
2-dTTP	1.035	0.838	0.717	0.453	0.929	0.655	0.788	0.461	1.099	0.814	0.549	0.327
2-oxoglutarate	3.615	2.880	2.248	1.606	5.075	4.390	2.951	2.134	2.983	2.379	2.027	1.455
2-phosphoglycerate	1.635	1.548	1.611	1.252	1.144	0.836	1.101	1.226	1.386	1.588	1.070	1.082
3-hydroxybutyrate	0.686	1.531	0.538	2.214	1.039	1.338	0.372	1.222	2.235	3.996	0.933	1.115
3-phosphoglycerate	2.379	1.667	1.540	1.382	1.512	0.954	1.012	1.567	2.097	1.682	1.049	1.159
4-hydroxyproline	9.704	3.867	10.750	3.136	2.131	2.425	4.012	2.977	11.181	11.012	9.354	4.658
5-oxoproline	2.894	6.179	2.716	3.050	2.284	4.124	2.291	2.808	2.863	2.630	3.435	4.221
6-phospho gluconate	1.172	1.452	1.254	0.813	0.975	1.137	1.028	1.235	0.989	1.032	1.003	0.847
acetyl-CoA	1.951	1.071	0.918	0.846	2.058	1.143	0.856	0.987	3.435	2.020	1.500	0.930
adenine	2.695	1.468	1.260	1.029	0.746	1.436	1.268	1.045	3.400	2.318	1.491	1.147
ADP	0.685	0.739	0.719	0.489	0.859	0.801	0.933	0.621	0.577	0.556	0.575	0.419
ADP-glucose	1.051	1.174	0.712	0.755	0.748	0.784	0.815	0.679	1.396	1.025	0.686	0.732
ADP-ribose	0.755	0.573	0.657	0.447	0.840	0.635	1.027	0.427	0.784	0.617	0.698	0.531
alanine	1.613	1.882	1.349	1.032	1.042	1.200	1.030	1.037	1.445	1.362	1.155	0.914
AMP	1.106	0.957	0.930	0.801	1.002	0.830	1.004	0.865	1.142	0.946	0.883	0.744
asparagine	1.948	1.504	2.342	0.817	1.360	1.275	1.495	0.881	1.733	1.477	1.982	0.944
aspartate	2.889	2.730	1.382	1.443	1.726	1.474	0.886	1.418	3.550	2.862	1.313	1.314
ATP	1.198	1.060	0.941	0.765	0.984	0.883	0.936	0.803	1.189	1.002	0.859	0.700
c-di-AMP	0.813	1.068	0.655	0.296	0.900	0.778	0.713	0.317	0.553	0.472	0.551	0.217
CDP	0.837	1.003	0.739	0.350	0.623	0.838	1.214	0.351	0.802	0.851	0.562	0.296
CDP-choline	0.839	0.866	0.617	0.199	0.783	0.859	0.807	0.353	0.850	0.561	0.597	0.206
CDP-ribitol	1.154	1.509	1.290	0.593	0.862	1.063	0.932	0.806	1.197	1.294	1.516	0.817
citrulline	1.146	1.112	1.216	1.241	0.403	0.655	0.745	1.126	0.583	0.747	1.097	1.192
CMP	0.506	0.957	0.654	0.322	0.401	0.866	1.424	0.441	0.403	0.536	0.706	0.239
CTP	1.065	1.103	0.839	0.379	0.788	0.813	0.779	0.386	0.918	0.875	0.610	0.269
cysteine	1.337	2.002	1.622	1.097	0.622	0.740	1.401	0.642	1.534	2.920	1.539	0.942
cystine	1.793	1.529	2.465	1.007	1.635	1.738	1.984	1.009	1.799	2.012	2.229	1.195
dihydroxyacetone phosphate	1.189	1.246	1.197	1.318	1.017	1.202	1.156	1.339	1.397	1.414	0.897	1.462
FAD	1.283	1.327	1.128	0.868	1.105	1.064	1.140	0.864	1.098	1.132	0.909	0.724
FMN	1.272	1.180	1.285	1.102	1.098	0.929	1.362	0.944	1.089	1.139	0.984	0.731
fructose	1.215	1.009	0.637	0.485	0.954	0.919	0.618	0.464	1.289	1.117	0.462	0.368

$FC = \frac{mean\ value\ t_x\ (treatment)}{mean\ value\ t_x\ (control)}$	cefotaxime				azithromycin				combination of cefotaxime and azithromycin			
	t_{15}	t_{30}	t_{60}	t_{90}	t_{15}	t_{30}	t_{60}	t_{90}	t_{15}	t_{30}	t_{60}	t_{90}
fructose-1,6-bisphosphate	1.327	1.491	1.442	1.963	1.259	1.385	1.256	2.084	1.502	1.304	1.268	1.903
fructose-6-phosphate	1.020	0.991	0.805	0.765	0.937	1.218	0.990	0.910	1.134	1.077	0.539	0.988
GDP	0.787	0.818	0.705	0.775	1.023	0.894	0.714	0.930	0.896	0.942	1.008	0.933
GlcNAc-6-P	0.832	0.637	0.694	0.526	1.378	1.024	1.037	0.674	0.793	0.574	0.516	0.403
glucose	2.524	1.504	3.349	0.972	1.246	1.097	1.617	0.830	3.160	2.542	2.887	1.223
glucose-6-phosphate	1.473	1.238	1.547	1.440	1.232	1.313	1.524	1.493	1.664	1.596	1.422	1.710
glutamate	2.948	3.403	3.974	1.527	1.696	1.720	1.688	0.898	1.832	1.728	2.405	1.024
glutamine	3.197	2.656	2.815	0.586	1.129	1.151	1.272	0.382	2.001	1.909	1.361	0.485
glycerol	1.310	0.938	0.883	0.895	0.827	0.929	1.018	0.958	1.577	1.339	0.773	0.948
glycerol-1-phosphate	1.366	1.261	1.204	1.281	1.078	1.148	1.333	1.069	1.886	1.740	1.091	1.432
glycine	3.345	2.080	1.800	0.938	1.349	1.256	1.023	0.942	3.578	2.720	1.310	0.966
GMP	1.036	1.172	1.071	0.658	0.841	0.936	0.849	0.325	0.916	1.068	0.768	0.331
GSSG	0.793	1.228	0.812	0.527	0.658	0.512	0.649	1.153	0.308	0.393	0.259	0.274
GTP	1.126	0.935	0.963	1.106	1.213	0.916	0.955	1.142	1.097	0.920	0.852	0.913
IMP	0.721	0.903	0.469	0.471	1.057	1.403	0.739	0.670	0.689	0.703	0.427	0.457
isoleucine	1.384	1.068	0.827	0.358	0.909	0.780	0.550	0.283	1.622	1.059	0.721	0.240
lactate	3.370	1.711	1.513	0.844	1.415	1.148	0.716	0.935	5.601	5.406	1.746	0.968
leucine	2.673	1.858	1.126	0.905	1.427	1.422	0.849	0.865	3.145	2.598	0.833	0.647
L-myo-inositol triphosphate	1.145	1.921	0.682	2.579	1.258	1.582	1.141	4.298	2.417	2.386	0.976	3.420
lysine	1.431	0.968	0.753	0.475	1.144	1.074	0.766	0.534	2.072	1.654	0.882	0.537
malonyl-CoA	1.632	1.477	0.538	0.496	1.348	1.343	0.734	0.498	2.859	2.142	0.527	0.366
methionine	2.635	2.040	1.492	1.152	2.330	1.618	1.140	1.030	3.094	2.667	1.193	1.024
NAD^+	0.867	0.945	0.837	0.644	0.912	0.879	1.011	0.705	1.020	0.978	0.859	0.725
$NADP^+$	1.848	1.849	1.891	2.091	1.094	1.130	1.314	2.110	1.919	1.797	1.819	2.081
ornithine	2.446	1.720	0.927	0.757	1.598	3.588	0.596	0.171	1.045	0.544	0.182	0.121
pantothenic acid	1.170	1.169	0.395	10.431	0.903	1.418	0.327	1.000	0.505	0.230	0.067	1.000
pantothenate-4-phosphate	1.031	1.512	1.234	5.083	0.862	1.066	0.904	2.833	0.238	0.176	0.185	0.515
phenylalanine	4.569	4.798	3.159	0.650	2.391	3.293	1.256	0.426	5.404	6.820	2.811	0.784
phenylpyruvate	1.792	1.407	1.070	1.468	1.166	1.015	1.044	1.257	2.384	2.255	0.957	1.705
phosphoenolpyruvate	3.361	2.146	1.570	1.705	2.134	1.247	1.047	2.039	3.905	3.759	1.048	1.462
proline	4.241	1.912	3.257	0.962	1.501	1.299	1.292	0.741	4.797	3.722	2.865	1.225
PRPP	1.422	1.301	1.002	1.011	1.459	1.153	0.817	0.895	1.686	1.327	0.863	0.705
pyruvate	2.173	1.617	1.011	0.768	1.368	1.078	0.956	0.863	2.862	2.839	1.175	0.758
ribose-5-phosphate	0.889	0.888	0.586	0.424	0.943	1.056	0.721	0.526	1.066	0.918	0.650	0.455
sedoheptulose-1,7-bisphosphate	1.258	1.186	1.183	1.134	1.371	1.297	1.276	1.441	1.203	1.001	0.976	1.117
sedoheptulose-7-phosphate	0.493	0.554	0.469	0.981	1.128	1.192	0.869	1.203	0.894	0.724	0.528	1.689
serine	2.335	1.526	1.485	1.277	1.241	1.201	1.011	1.156	3.271	2.695	1.432	1.299
succinate	2.781	2.677	2.260	1.897	1.584	1.551	1.485	1.284	2.617	2.150	1.686	1.470
threonine	2.226	1.953	1.040	0.956	1.344	1.283	0.805	1.018	2.706	2.341	0.846	0.876

$FC = \frac{mean\ value\ t_x\ (treatment)}{mean\ value\ t_x\ (control)}$	cefotaxime				azithromycin				combination of cefotaxime and azithromycin			
	t_{15}	t_{30}	t_{60}	t_{90}	t_{15}	t_{30}	t_{60}	t_{90}	t_{15}	t_{30}	t_{60}	t_{90}
tyrosine	3.054	1.509	2.964	0.772	1.435	1.048	1.505	0.669	3.669	2.394	2.674	0.846
UDP	1.193	1.302	1.006	0.598	0.857	0.741	0.907	0.582	1.255	1.137	0.965	0.592
UDP-GlcNAc/-GalNAc	1.743	1.309	1.044	0.610	1.276	1.040	0.929	0.635	1.885	1.347	0.832	0.499
UDP-GlcNAc-enolpyruvate	1.143	0.855	0.662	0.278	1.132	0.687	0.569	0.265	0.934	0.629	0.491	0.253
UDP-glucose/galactose	1.581	1.293	1.107	0.681	1.114	0.908	0.916	0.696	1.891	1.347	0.997	0.603
UDP-MurNAc	1.238	1.308	1.636	0.941	1.188	0.969	1.017	0.820	1.670	1.331	1.845	1.184
UDP-MurNAc-Ala	2.222	2.394	1.543	1.162	1.529	1.205	0.893	0.952	3.338	2.413	1.583	2.068
UDP-MurNAc-Ala-Glu-Lys	0.644	1.147	1.112	0.256	0.506	1.037	0.978	0.261	0.807	1.037	2.885	0.811
UDP-MurNAc-Ala-Glu-Lys-Ala-Ala	1.080	1.238	1.021	0.528	0.848	1.190	0.879	0.520	0.996	1.672	3.621	2.722
UMP	1.075	0.994	0.923	0.564	0.902	0.871	1.037	0.545	1.041	0.837	0.710	0.432
uracil	2.423	1.253	1.524	1.068	0.738	0.925	1.139	0.859	2.640	1.884	1.589	1.447
UTP	1.272	1.082	0.914	0.572	1.046	0.885	0.907	0.538	1.229	0.900	0.657	0.438
valine	1.668	1.079	1.621	1.232	2.686	1.281	1.292	0.989	2.448	1.650	1.311	1.251
XMP	0.426	0.227	0.252	0.364	1.454	0.529	0.607	0.610	0.758	0.281	0.235	0.454

$FC = \frac{mean\ value\ t_x\ (treatment)}{mean\ value\ t_x\ (control)}$	moxifloxacin				teixobactin-Arg10			
	t_{15}	t_{30}	t_{60}	t_{90}	t_{15}	t_{30}	t_{60}	t_{90}
1,3-bisphosphoglycerate	1.327	0.911	1.064	0.860	1.183	0.949	0.997	0.616
2-dADP	1.608	1.651	1.826	1.585	0.831	0.848	0.679	0.395
2-dATP	1.480	1.525	1.644	1.312	0.888	0.998	0.746	0.427
2-dCTP	0.513	0.555	0.659	0.711	0.992	0.915	0.544	0.231
2-dTDP	0.654	0.607	0.736	0.535	0.635	0.556	0.387	0.149
2-dTMP	1.179	1.234	1.245	1.143	1.221	1.206	0.514	0.264
2-dTTP	1.078	0.909	1.022	0.640	1.256	1.004	0.662	0.245
2-oxoglutarate	2.902	0.987	1.947	2.048	1.593	0.877	0.688	1.155
2-phosphoglycerate	1.372	1.776	1.837	1.490	1.025	1.133	1.700	0.722
3-hydroxybutyrate	0.840	1.567	4.912	0.811	0.116	1.161	1.546	2.412
3-phosphoglycerate	1.873	1.869	1.667	1.885	1.425	1.292	1.406	0.792
4-hydroxyproline	3.905	5.090	7.083	1.447	10.747	8.377	9.141	3.357
5-oxoproline	3.268	5.111	3.534	2.117	3.805	3.985	2.518	1.746
6-phospho gluconate	1.192	1.385	1.248	1.475	0.861	0.985	0.647	0.359
acetyl-CoA	1.390	0.795	1.161	0.855	2.579	1.281	1.111	0.863
adenine	2.650	2.163	1.739	1.554	3.928	3.111	1.875	1.681
ADP	0.821	0.830	0.953	0.743	0.627	0.566	0.484	0.296
ADP-glucose	1.270	1.154	1.146	1.333	1.702	1.209	0.697	0.788
ADP-ribose	0.630	0.561	0.884	0.438	0.515	0.537	0.756	0.392
alanine	1.228	1.373	1.168	1.085	1.076	1.112	0.692	0.412
AMP	1.183	1.035	1.185	0.876	1.095	0.919	0.779	0.514
asparagine	1.395	1.685	1.599	0.789	1.685	1.690	1.431	0.694
aspartate	2.015	1.792	1.200	1.174	2.434	2.002	0.967	0.622
ATP	1.156	1.073	1.123	0.858	1.181	1.001	0.770	0.495
c-di-AMP	0.864	0.847	0.750	0.584	0.844	0.766	0.309	0.119
CDP	1.518	2.028	2.439	1.422	0.928	0.892	0.408	0.128
CDP-choline	1.414	1.871	2.247	0.989	1.145	0.813	0.304	0.104
CDP-ribitol	1.743	2.395	2.327	1.535	1.348	1.615	1.485	0.827
citrulline	1.292	1.864	0.878	1.334	1.194	1.961	1.231	1.122
CMP	0.859	1.491	1.274	1.128	0.443	0.661	0.340	0.179
CTP	1.501	1.918	2.200	1.206	1.086	1.020	0.498	0.160
cysteine	0.617	1.963	0.844	0.457	1.314	2.353	1.771	0.737
cystine	0.778	1.641	1.086	0.749	1.351	2.595	2.686	0.920
dihydroxyacetone phosphate	1.203	1.402	1.168	0.921	1.100	1.178	1.042	0.786
FAD	1.113	1.039	1.057	0.897	1.150	1.095	0.934	0.719
FMN	1.223	1.024	1.232	0.878	1.237	1.104	0.919	0.660
fructose	1.040	1.088	0.776	0.603	1.114	1.866	0.586	0.404

$FC = \frac{mean\ value\ t_x\ (treatment)}{mean\ value\ t_x\ (control)}$	moxifloxacin				teixobactin-Arg10			
	t_{15}	t_{30}	t_{60}	t_{90}	t_{15}	t_{30}	t_{60}	t_{90}
fructose-1,6-bisphosphate	1.277	1.318	1.219	1.394	1.162	1.099	0.768	0.895
fructose-6-phosphate	0.927	1.166	0.947	0.602	0.822	1.001	0.988	0.739
GDP	1.489	1.728	2.172	3.514	0.903	0.966	0.658	0.772
GlcNAc-6-P	0.875	0.681	0.789	0.587	0.696	0.497	0.466	0.308
glucose	1.399	1.547	2.014	0.583	3.019	3.213	2.352	1.099
glucose-6-phosphate	1.154	1.401	1.368	1.061	1.379	1.460	1.611	1.305
glutamate	1.765	1.797	2.114	1.285	1.405	1.292	0.640	0.306
glutamine	1.459	1.891	2.003	0.404	2.164	2.596	0.778	0.420
glycerol	1.393	1.323	1.352	1.151	1.497	1.183	1.071	0.966
glycerol-1-phosphate	1.667	2.102	1.740	1.593	1.882	1.519	1.715	1.150
glycine	1.629	1.946	1.405	0.958	2.864	2.737	0.987	0.561
GMP	0.938	0.952	1.092	0.547	0.806	0.715	0.622	0.224
GSSG	1.166	0.590	0.432	0.832	0.806	0.679	0.634	0.449
GTP	1.405	1.367	2.038	3.234	0.984	0.859	0.642	0.612
IMP	0.826	0.722	0.493	1.080	0.581	0.474	0.237	0.202
isoleucine	0.743	0.833	0.829	0.418	1.140	1.478	0.404	0.157
lactate	1.563	1.561	1.229	1.219	5.470	4.742	1.270	0.912
leucine	1.469	1.791	1.314	0.798	2.549	2.364	0.745	0.394
L-myo-inositol triphosphate	1.593	1.422	1.131	1.297	1.417	1.292	0.630	1.403
lysine	1.162	1.203	0.931	0.667	1.676	1.351	0.856	0.507
malonyl-CoA	1.660	1.199	0.851	0.680	2.325	1.464	0.418	0.291
methionine	1.996	2.117	1.759	1.220	2.653	2.523	1.086	0.583
NAD^+	0.941	0.994	1.030	1.008	0.789	0.690	0.748	0.496
$NADP^+$	1.750	1.784	2.008	2.273	1.977	1.928	1.830	1.805
ornithine	0.864	1.189	0.583	0.095	1.347	1.327	0.079	0.074
pantothenic acid	1.329	1.416	0.124	1.000	0.819	0.836	0.143	1.000
pantothenate-4-phosphate	1.522	1.762	0.276	1.957	0.778	0.770	0.266	0.883
phenylalanine	2.820	4.736	2.880	0.584	5.066	7.944	2.403	0.378
phenylpyruvate	1.723	1.441	1.253	1.337	2.595	1.909	1.097	1.117
phosphoenolpyruvate	2.550	1.782	1.617	2.442	2.828	2.351	1.477	0.837
proline	1.714	1.903	2.023	0.681	4.419	4.764	2.229	0.990
PRPP	0.954	0.728	0.651	0.741	1.396	1.002	0.632	0.375
pyruvate	2.242	1.670	1.278	1.160	2.450	2.357	1.188	0.670
ribose-5-phosphate	1.088	0.866	0.636	0.411	0.926	0.694	0.689	0.359
sedoheptulose-1,7-bisphosphate	1.136	1.100	1.106	0.893	0.953	0.812	0.668	0.582
sedoheptulose-7-phosphate	0.533	0.402	0.460	0.431	0.769	0.639	0.519	0.839
serine	1.340	1.590	1.293	1.007	2.622	2.469	1.319	0.878
succinate	1.439	0.947	1.397	1.541	1.448	1.277	0.442	0.678
threonine	1.726	1.782	1.144	0.963	1.994	1.728	0.685	0.456

$FC = \frac{mean\ value\ t_x\ (treatment)}{mean\ value\ t_x\ (control)}$	moxifloxacin				teixobactin-Arg10			
	t_{15}	t_{30}	t_{60}	t_{90}	t_{15}	t_{30}	t_{60}	t_{90}
tyrosine	1.839	1.718	2.816	0.743	3.519	3.066	2.618	0.730
UDP	2.197	2.297	2.162	1.357	1.305	1.147	0.772	0.360
UDP-GlcNAc/-GalNAc	1.862	1.723	1.643	0.913	1.841	1.544	0.928	0.432
UDP-GlcNAc-enolpyruvate	1.565	1.385	1.121	0.401	1.509	1.350	0.871	0.394
UDP-glucose/galactose	1.499	1.347	1.382	0.893	1.693	1.411	1.019	0.478
UDP-MurNAc	1.415	1.188	1.170	0.749	2.175	2.124	2.748	2.448
UDP-MurNAc-Ala	2.282	1.284	1.365	0.706	2.980	1.985	1.087	0.764
UDP-MurNAc-Ala-Glu-Lys	0.760	0.821	0.928	0.416	0.763	1.327	4.783	1.847
UDP-MurNAc-Ala-Glu-Lys-Ala-Ala	1.047	1.159	1.027	0.422	1.142	2.283	6.758	5.605
UMP	1.163	1.255	1.289	0.761	0.921	0.864	0.725	0.311
uracil	2.094	2.039	2.061	1.396	3.084	2.902	2.183	1.534
UTP	1.528	1.473	1.486	0.874	1.233	0.972	0.628	0.303
valine	1.025	1.013	2.194	1.003	1.824	1.519	1.304	0.693
XMP	0.060	0.030	0.190	0.512	0.402	0.227	0.351	0.327

TabS.5: Metabolites measured by GC-MS and identified by database alignment.

	Formula	RT	Score (libary)	Libary
adenine	$C_5H_5N_5$	29.101	82.51	Fiehn.L
citrulline	$C_6H_{13}N_3O_3$	28.325	85	Fiehn.L
glycerol	$C_3H_8O_3$	15.002	91.63	Fiehn.L
glycerol-1-phosphate	$C_3H_9O_6P$	26.997	89.31	Fiehn.L
isoleucine	$C_6H_{13}NO_2$	15.621	81.6	Fiehn.L
uracil	$C_4H_4N_2O_2$	16.707	88.94	NIST17.L

TabS.6: Calculated and detected masses of metabolites measured by HPLC-MS and identified by database alignment (Human Metabolome Database).

	$[M-H]^-$ calculated	$[M-H]^-$ detected
1,3-bisphosphoglycerate	264.951	264.954
2-dADP	410.027	410.030
2-dATP	489.993	489.998
2-dCTP	465.982	465.987
2-dTDP	401.015	401.019
2-dTMP	321.049	321.052
2-dTTP	480.982	480.982
acetyl-CoA	808.118	808.125
ADP-glucose	588.074	588.071
ADP-ribose	598.064	558.068
CDP-choline	488.108	487.104
CDP-ribitol	536.069	536.068
cyclic-di-AMP	657.097	657.097
FAD	784.149	784.156
flavin mononucleotide (FMN)	455.098	455.101
GlcNAc-6-P	300.048	300.052
glutathione disulfide (GSSG)	611.144	611.149
ITP	506.972	506.976
L-myo-inositol triphosphate	418.954	418.959
NAD^+	662.101	662.100
$NADP^+$	742.068	742.067
pantothenate-4-phosphate	298.076	298.072
pantothenic acid	218.103	218.106
sedoheptulose-1,7-bisphosphate	368.999	369.003
sedoheptulose-7-phosphate	289.032	289.039
UDP-GlcNAc-enolpyruvate	676.079	676.079
UDP-MurNAc	678.095	678.094
UDP-MurNAc-Ala	749.132	749.138
UDP-MurNAc-Ala-Glu-Lys	502.631 $[M-2H]^{2-}$	502.631 $[M-2H]^{2-}$
UDP-MurNAc-Ala-Glu-Lys-Ala-Ala	573.668 $[M-2H]^{2-}$	573.668 $[M-2H]^{2-}$
