# Supplementary material for: Exploring metabolic adaptation of Streptococcus pneumoniae to antibiotics
Source: J Antibiot (Tokyo). 2020 Mar 24;73(7):441–54. doi: 10.1038/s41429-020-0296-3 (PMC7292801; doi:10.1038/s41429-020-0296-3)
Supplement: Supplementary file 2 — Supplemental Figures Leonard et al. [file 41429_2020_296_MOESM2_ESM.pdf]

Supplemental material

Exploring metabolic adaptation of Streptococcus pneumoniae to antibiotics

Anne Leonard[a], Kevin Möhlis[a], Rabea Schlüter[b], Edward Taylor[c], Michael Lalk[a], Karen Methling[a*]

[a] University of Greifswald, Institute for Biochemistry, Metabolomics, Felix-Hausdorff-Str. 4, 17489 Greifswald, Germany

[b] University of Greifswald, Imaging Center of the Department of Biology, F.-L-Jahn-Str. 15, 17489 Greifswald, Germany

[c] University of Lincoln, School of Life Sciences, Green Lane, LN67DL, Lincoln, England

[*]Corresponding author. Tel.: + 49 (0)3834 420-4167; fax: + 49 (0)3834 420-4479.
E-mail address: methling@uni-greifswald.de (K. Methling)

Keywords: *Streptococcus pneumoniae*, metabolism, antibiotics, antimicrobial substance, adaptation

Supplementary figures

Table of contents:

Fig.S1: Minimal inhibitory concentrations of antibiotic compounds in *S. pneumoniae* TIGR4*Δcps*

Fig.S2: Colony forming units of *S. pneumoniae* TIGR4*Δcps* under antibiotic stress conditions

Fig.S3: Transmission electron micrographs of *S. pneumoniae* TIGR4*Δcps* cells

Fig.S4: Uptake of arginine and accumulation of ornithine extracellularly after addition of antibiotic compounds at t_{90}

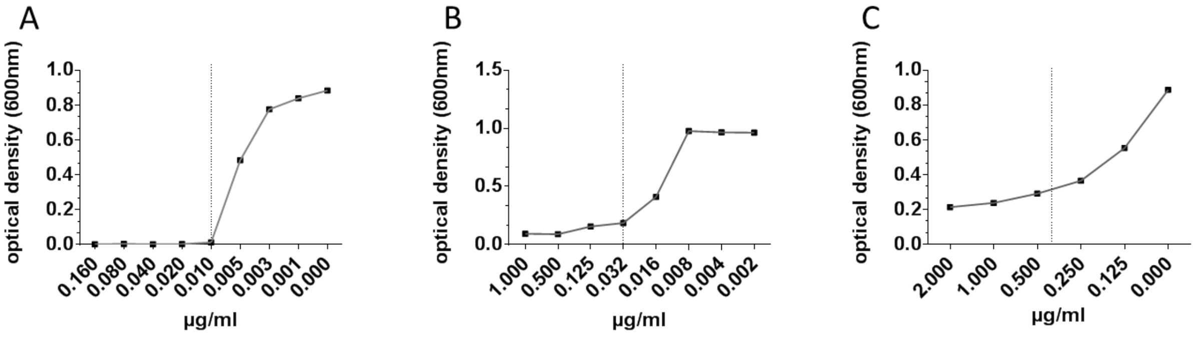

Fig.S1: Minimal inhibitory concentrations of antibiotic compounds in *S. pneumoniae* TIGR4*Δcps*. Cefotaxime (A) (MIC 0.01 µg/ml); azithromycin (B) (MIC 0.032 µg/ml); moxifloxacin (C) (MIC 0.4 µg/ml).

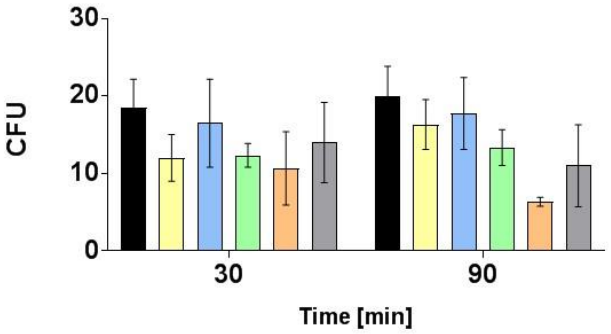

Fig.S2: Colony forming units of *S. pneumoniae* TIGR4*Δcps* under different stress conditions: control (black), cefotaxime (yellow), azithromycin (blue), combination of cefotaxime and azithromycin (green), moxifloxacin (orange) and teixobactin-Arg10 (grey).

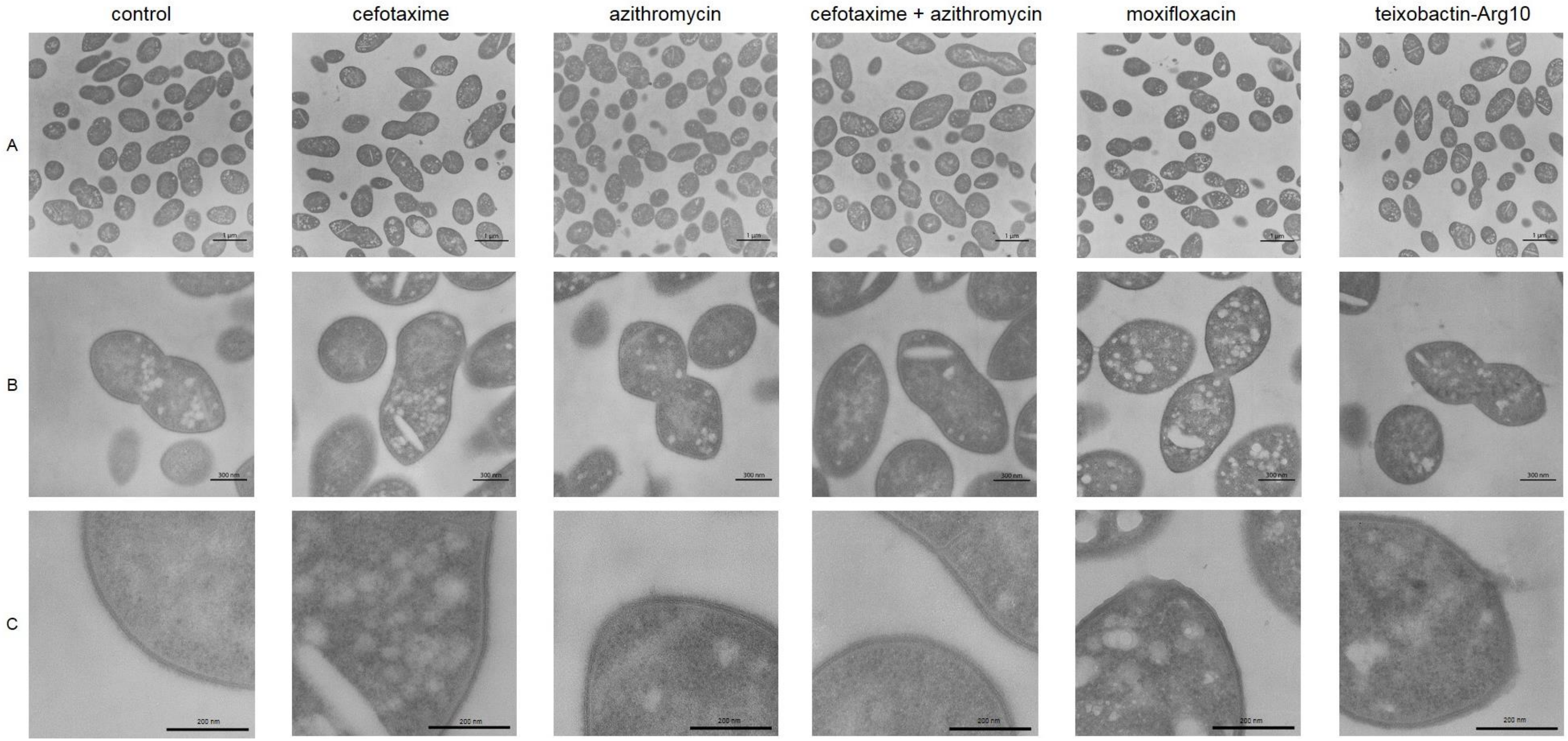

Fig.S3: Transmission electron micrographs of *S. pneumoniae* TIGR4*Δcps* cells grown in modified RPMI medium under control conditions and after addition of the antibiotic compounds cefotaxime, azithromycin, combination of cefotaxime and azithromycin, moxifloxacin, and teixobactin-Arg10. Magnification = 10.000fold, scale bar = 1 µm, preparation method B (A), magnification = 35.970fold, scale bar = 300 nm, preparation method A (B), magnification = 60.000fold, scale bar = 200 nm, preparation method A (C)

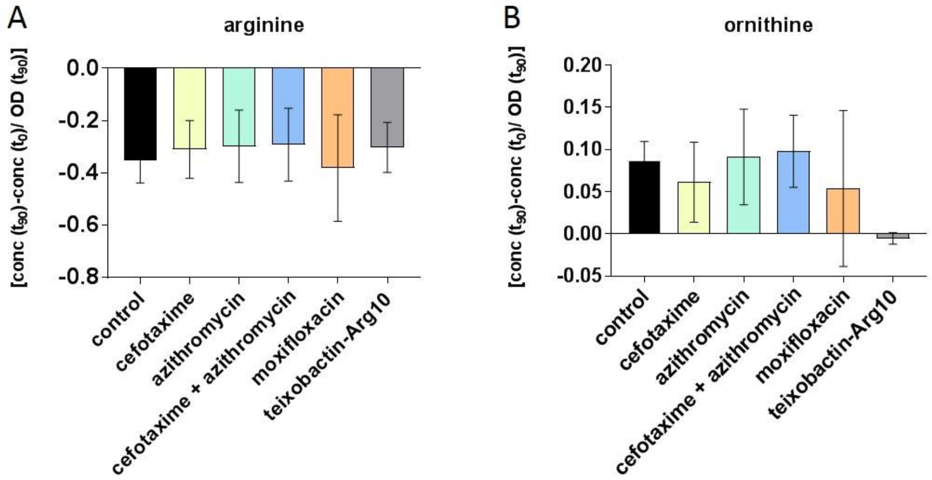

Fig.S4: Uptake of extracellular arginine (A) and accumulation of ornithine extracellularly (B) under control conditions and after addition of antibiotic compounds at t_{90}. Columns are colored according to the antibiotic stresses: control (black), cefotaxime (yellow), azithromycin (blue), combination of cefotaxime and azithromycin (green), moxifloxacin (orange) and teixobactin-Arg10 (grey). Data are shown as mean values ± standard derivation. (n=4-5)
